# Multiomic Metabolic Enrichment Network Analysis Reveals Metabolite–Protein Physical Interaction Subnetworks Altered in Cancer

**DOI:** 10.1016/j.mcpro.2021.100189

**Published:** 2021-12-20

**Authors:** Benjamin C. Blum, Weiwei Lin, Matthew L. Lawton, Qian Liu, Julian Kwan, Isabella Turcinovic, Ryan Hekman, Pingzhao Hu, Andrew Emili

**Affiliations:** 1 Center for Network Systems Biology, Boston University, Boston, Massachusetts, USA; 2 Department of Biochemistry, Boston University School of Medicine, Boston, Massachusetts, USA; 3 Departments of Biochemistry and Medical Genetics, University of Manitoba, Winnipeg, Manitoba, Canada; 4 Department of Biology, Boston University, Boston, Massachusetts, USA

**Keywords:** networks, cancer, systems biology, proteomics, multiomic, CCLE, Cancer Cell Line Encyclopedia, CPTAC, Clinical Proteomic Tumor Analysis Consortium, DMEM, Dulbecco’s modified Eagle’s medium, FBS, fetal bovine serum, FDR, false discovery rate, GSEA, gene set enrichment analysis, HPLC, high-pressure liquid chromatography, MOMENTA, multiomic metabolic enrichment network analysis, MS, mass spectrometry, nLC, nanoflow liquid chromatography, PANAMA, proteomic and nanoflow metabolomic analysis, PPI, protein–protein interaction, PTM, posttranslational modification, QC, quality control, TMT, tandem mass tag

## Abstract

Metabolism is recognized as an important driver of cancer progression and other complex diseases, but global metabolite profiling remains a challenge. Protein expression profiling is often a poor proxy since existing pathway enrichment models provide an incomplete mapping between the proteome and metabolism. To overcome these gaps, we introduce multiomic metabolic enrichment network analysis (MOMENTA), an integrative multiomic data analysis framework for more accurately deducing metabolic pathway changes from proteomics data alone in a gene set analysis context by leveraging protein interaction networks to extend annotated metabolic models. We apply MOMENTA to proteomic data from diverse cancer cell lines and human tumors to demonstrate its utility at revealing variation in metabolic pathway activity across cancer types, which we verify using independent metabolomics measurements. The novel metabolic networks we uncover in breast cancer and other tumors are linked to clinical outcomes, underscoring the pathophysiological relevance of the findings.

## Highlights

• Integrating protein interaction data with metabolic models expands multiomic mapping.
• Proteomic profiling of tumors and cell lines reveals altered metabolic-related signatures.
• Metabolite measurements validate pathway alterations in cancer cell lines and tumors.

## Graphical Abstract

**Figure:**
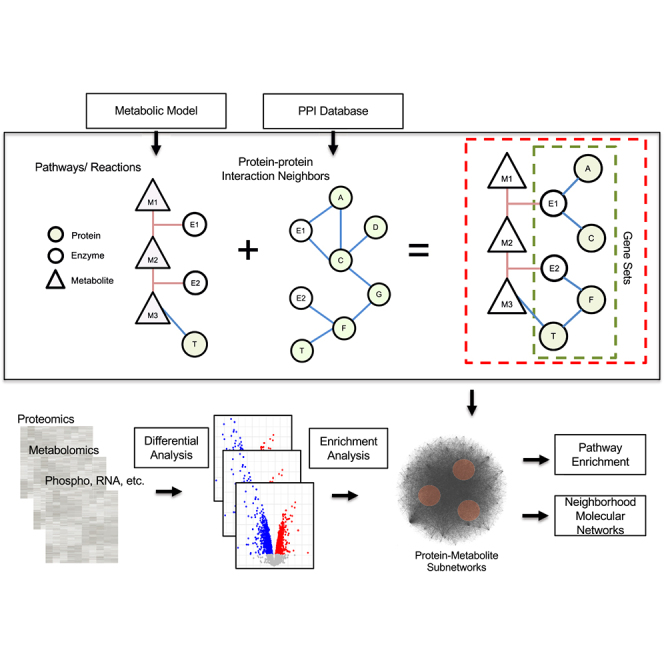

## In Brief

Metabolism is recognized as an important driver of complex diseases, but global metabolite profiling remains a challenge. Protein expression is a poor proxy because pathway enrichment models provide an incomplete mapping between the proteome and metabolism. We developed MOMENTA, a multiomic network approach for interrogating metabolic pathways from proteomics data. Analysis of data from cancer cell lines and human tumors reveals metabolic network rewiring and oncogene connections. The metabolic networks altered in cancer are linked to clinical outcomes.

Regulation of metabolism through rewiring of biochemical pathways occurs in response to physiological and pathobiological signals, and dysregulation is increasingly linked to progression of complex diseases, including cancer (1). Cellular metabolic pathways are controlled by changes in enzyme (catalytic subunit) and cofactor (regulatory subunit) expression, by protein–protein interactions (PPI) that constrain component localization/compartmentalization, by posttranslational modification(s) (PTM; *e.g.*, phosphorylation), and local substrate/product concentrations, as well as through allosteric regulation (*e.g.*, ligand binding), motivating the need to leverage functional proteomic and metabolomic information together for in-depth interrogation of cellular metabolic regulation (2, 3, 4, 5, 6).

Massive genome and proteome profiling efforts have been invaluable in elucidating many of the molecular drivers of cancer (7, 8). These studies and the subsequent analyses have contributed to our understanding of this complex disease and identified many potentially actionable therapeutic hypotheses. Recent efforts have also turned to global profiling of metabolites; however, the cohort sizes and number of features identified are much smaller, limiting the degree to which we can expect findings to be generalizable (9). Despite this, there remains a significant interest in more thoroughly understanding the complete degree to which metabolism is altered in cancer types in order to identify potential vulnerabilities or dependencies that may lead to actionable insights.

Unbiased characterization of cellular pathway activity depends on comprehensive molecular profiling. Mass spectrometry (MS) is a technology of choice for studying changes in the abundance and interactions of proteins, PTMs, and small molecules in response to physiological cues and pathological stresses (10, 11). However, given their chemical diversity, global experimental identification of metabolites remains challenging (12), and relatively few laboratories proficient in proteomics engage in direct metabolomic profiling. In contrast, metabolic networks and models are some of the most comprehensively annotated models across all biological processes (13, 14), and pathway annotations from metabolic models form much of the basis for interpreting and understanding metabolism in biological systems (15). More informed strategies to better infer metabolic changes from phospho/proteomic profiles, and/or transcriptomic datasets, are therefore warranted. Numerous omics data integration tools have been devised to examine correlated changes between various molecular layers in cell systems (*e.g.*, (16)), including many that infer changes in metabolic pathway activity (*e.g.*, enzyme-mediated metabolic reactions) and exploit *a priori* knowledge of protein–protein and protein–metabolite interactions to integrate disparate data types (17, 18, 19, 20).

One strategy for interpreting proteomic profiles from a metabolism perspective is to impose a network architecture of physical associations over a metabolic model in order to flesh out functional associations missed by traditional curation efforts. Network models are especially useful for integrating disparate biomolecular data types (21, 22), potentially illuminating unexpected mechanisms regulating biochemical pathways. The biophysical properties of networks (*e.g.*, connecting edges, network propagation) enable the study of dynamic biomolecular systems that respond to perturbations. By representing metabolites, enzymes, and their other cellular interaction partners (*e.g.*, ligands, regulatory subunits) as nodes, and reactions and interactions as linking edges, networks provide a unifying mechanistic framework to systematically explore and discover unexpected molecular relationships and functional dependencies (23). Combining metabolic models with the results from large-scale PPI surveys can also aid in the identification of small-molecule compound by MS (24, 25).

Traditionally, comparative (cases *versus* controls) proteomic surveys utilize some form of enrichment analysis to find functional trends among a set of observed differential molecular features. Such approaches fail to fully leverage metabolic models, which currently have annotated associations between metabolites, enzymes, regulators/cofactors, transporters and their respective small-molecule ligands that encompass only about 1200 to 1500 distinct gene products (15), covering only a small fraction (∼6%) of the expressed human proteome (26). This limits the utility of gene-centric (proteomic, transcriptomic) profiling data to faithfully interrogate and predict changes in metabolic pathway activity. To address this gap, we adapted previous models of metabolic network and PPI integration to generate gene sets for use in gene set enrichment analysis (GSEA) with proteomic data (17). Specifically, this implementation, which we call Multiomic Metabolic Enrichment Network Analysis (MOMENTA) connects prior physical (PPI networks) and functional information (pathway annotation from metabolic models) to interrogate metabolic pathways in a comparative study (*i.e.*, disease *versus* control samples) using proteomic (or transcriptomic) data types. This network principle provides a unifying foundation to interpret changes in proteomic and metabolic activity at a biochemical pathway level since the functional relationships are centered on metabolic pathways, derived from the same metabolic model for both the proteomic and metabolomic analysis. Statistical enrichment analysis with MOMENTA on input differential protein (or gene) derived measurements using a functional model that extends metabolic pathways with reliable and scored PPI information elucidates biochemical pathway activity beyond mere correlations or primary pathway annotations.

As proof of concept and validation of our implementation, we applied MOMENTA to examine alterations in cell metabolism based on the quantitative proteomic profiles of cultured breast cancer cells following a controlled metabolic perturbation, before examining proteomic profiling of a broader compendium of cancer cell lines, and in human tumor-derived clinical samples. In each case, we obtain systems-level insights into the biochemical processes impacting metabolic pathway activity and cross talk beyond existing traditional approaches, which we were able to independently confirm using direct metabolic measurements. These case studies illustrate the utility of MOMENTA for integrating proteomic data with metabolomic models using a rigorously inferred network scaffold to generate more encompassing biochemical pathway descriptions that both leverage and contribute to improved cross-mapping between metabolites, enzymes, and their physical and functional interactions that are dynamically altered in a pathophysiological context.

## Experimental Procedures

### Experimental Design and Statistical Rationale

For cell culture proteomic and metabolomic analysis, cells were grown and analyzed in replicates of 5 and 6, respectively, for each of two conditions (with and without glucose). Differential analysis and comparison between groups are performed using a *t* test with adjustment made with for multiple testing (*e.g.*, Benjamini–Hochberg). For all studies, pathway-level findings are validated with orthogonal data sets.

### Cancer Cell Line Culture

MDA-MB-231, a human breast cancer–derived cell line, was obtained from the American Type Culture Collection (ATCC). Cells were maintained in Dulbecco’s modified Eagle’s medium (DMEM) without pyruvate containing 4.5 g/l of glucose and L-glutamine (Gibco), supplemented with 10% heat inactivated fetal bovine serum (FBS; Hyclone) and 100 units/ml penicillin-100 μg/ml streptomycin (Hyclone) in a humidified incubator at 37 °C in 5% CO_2_. Cells were then either continued to be cultured in the same medium or switched to a no (0%) glucose condition (DMEM containing L-glutamine but without glucose or pyruvate) for 48 h before harvesting.

### Protein Digestion

After pelleting, cells were resuspended in 100 μl of 8 M urea, containing protease inhibitors (cOmplete Protease Inhibitor Cocktail; Roche) and phosphatase inhibitors (PhosStop; Roche). After brief sonication on ice, the lysates were reduced with addition of dithiothreitol to a final concentration of 5 mM for 60 min at room temperature, followed by alkylation with the addition of iodoacetamide (5 mM) and incubation at room temperature for 30 min in the dark. The samples were then diluted with 50 mM ammonium bicarbonate to bring the urea concentration below 1 M. Proteins were digested overnight with sequence-grade (Thermo Scientific) trypsin (1:50 enzyme to protein ratio) at 37 °C followed by the addition of formic acid to 1% (v/v). The resulting peptides were desalted using a C18 cartridge as per the manufacturer’s instructions (Thermo Scientific).

### TMT Peptide Labeling

Peptide quantification was determined by Pierce quantitative colorimetric assay (Thermo Scientific). For each sample, 100 μg of peptide was resuspended in 0.1 M triethylammonium bicarbonate (TEAB) and incubated with a tandem mass tag (TMT) 10-plex isobaric labeling reagent (0.8 mg Thermo Scientific). The ratio of TMT to substrate was 0.4 mg reagent to 0.1 mg peptide. The reaction was carried out for 1 h at room temperature and quenched using 5% (v/v) hydroxylamine for 15 min. Equal amounts of each sample were combined in a new tube and desalted using a C18 Tip.

### High-pH Reverse-Phase Peptide Fractionation

The labeled peptide mixtures (1 mg) were fractionated using a Waters XBridge BEH C18 column (3.5 μm, 4.6 × 250 mm) on an Agilent 1100 HPLC system operating at a flow rate of 0.45 ml/min with two buffer lines: buffer A (consisting of 0.1% ammonium hydroxide-2% acetonitrile-water) and buffer B (consisting of 0.1% ammonium hydroxide-98% acetonitrile, pH 9). Peptides were separated by a gradient from 0% to 10% B in 5 min, followed by a linear increase to 30% B in 23 min, 60% B in 7 min, and then 100% in 8 min and continued for 5 min. The 48 fractions collected were combined into 12 and evaporated to dryness in a vacuum concentrator. Two micrograms of peptide from each fraction was reconstituted in 1% formic acid and kept at −80 °C until analyzed by the nLC-MS/MS system.

### Titanium Dioxide (TiO2) Enrichment of Fractionated Phosphopeptides

TiO2-coated magnetic beads (GL Sciences, Titansphere Phos-TiO) were used to enrich phosphopeptides obtained from combined HPLC fractions. Beads (5 μl/mg) were preconditioned with DHB buffer (consisting of 6% TFA, 5 mM KH2PO4, 80% ACN, 20 mg/ml 2,5-dyhydroxybenzoic acid) for 15 min, then incubated with the peptide mixtures, resuspended in 500 μl of DHB buffer (10:1 bead to peptide ratio, w/w), for 30 min with shaking. Beads were washed in steps: 1% TFA-80% ACN, followed by 1% TFA-50% ACN, and 1% TFA-10% ACN twice, then the supernatant was discarded to remove abundant contaminants. The phosphopeptides were eluted by 5% NH_4_OH with 25% ACN and dried by speed vac before nLC-MS analysis.

### Nanoflow LC-MS/MS of Proteomics and Phosphoproteomics

Total peptides and bead-enriched phosphopeptides from each HPLC fraction were individually loaded onto a C18 trap column (3 μm, 75 μm × 2 cm, Thermo Scientific), connected in-line to a C18 analytical EasySpray (Thermo Scientific) column (2 μm, 75 μm × 50 cm) using the EasyLC 1200 system (Thermo Scientific) in a column oven at 55 °C. The nanoflow gradient consisted of buffer A (composed of 2% (v/v) ACN with 0.1% formic acid) and buffer B (consisting of 80% (v/v) ACN with 0.1% formic acid). For protein analysis, nLC was performed for 180 min at a flow rate of 250 nl/min, with a gradient of 2% to 8% B for 5 min, followed by 8% to 20% B for 96 min, then 20% to 35% for 56 min, 35% to 98% B for 3 min, 98% buffer B for 3 min, followed by column recycling with 100% to 0% B for 3 min, and finishing with 5% B for 14 min. Peptides were directly ionized from a nanospray ion source into an online Q-Exactive HF (QE-HF) mass spectrometer (Thermo Scientific) operated in a data-dependent data acquisition mode.

The Q-Exactive HF was run using a ddMS2 top ten scans acquired per single profile mode full-scan mass spectrum using HCD fragmentation. Full MS spectra were collected at a resolution of 120,000 with an AGC of 3e^6^ or maximum injection time of 60 ms and a scan range of 350 to 1650 m/z. MS2 scans were performed at 45,000 resolution, with an ion-packet setting of 2e^4^ for AGC, maximum injection time of 90 ms and using 33% total normalized collision energy. Source ionization parameters were optimized with the spray voltage at 2.1 kV, transfer temperature at 275 °C. Dynamic exclusion was enabled for 40 s.

For the phosphopeptide analysis, the metal-bead enriched peptide fractions were loaded onto a 50 cm C18 column. nLC was performed for 90 min at a flow rate of 250 nl/min, with a gradient of 2% to 6% B for 5 min, followed by a 6% to 20% B for 39 min, a 20% to 35% gradient for 23 min, and a 35% to 98% B gradient for 3 min, 98% buffer B for 3 min, 100% to 0% gradient of B for 3 min, and finishing with 5% B for 14 min. The QE-HF was operated using a top six scan ddMS2 acquisition mode, with a maximum ms2 injection time of 400 ms.

### Proteome Data Analysis

For the *in vitro* cancer cell line data analysis, raw files from five (5) biological replicates for each condition were processed by MaxQuant (version 1.6) under standard settings using the UniProt Reviewed (Swiss-Prot) Human database with 20,443 entries (accessed July 2019). The extracted MS/MS spectra were searched against a both forward (native) and reversed (decoy) sequences, with protein identification allowing for two missed trypsin cleavage sites, variable modifications of methionine oxidation, N-terminal acetylation, and (for the phosphoproteomic data) protein phosphorylation at S, T, and Y residues. Carbamidomethylation of cysteine residues was set as a fixed modification. Matched ion tolerances of 20 and 6 ppm were set for the first and second searches, respectively. Candidate peptides, proteins, and phosphosite identifications were filtered based on a stringent, empirically controlled 1% false discovery rate (FDR) threshold, using a 2 min window to match identifications between runs. Phosphosites with a localization probability less than 0.7 were removed. Relative quantification was determined based on the TMT 10 plex reporter ion intensities measured by MS2.

### Metabolite Extraction and Cleanup

After pelleting, MDA-MB-231 cells (∼15 million) were incubated with 1 ml cold MeOH:ACN:H2O (40:40:20, v/v) solvent with vortexing for 30 s, followed by incubation in liquid nitrogen for 1 min. The samples were then allowed to thaw on ice followed by sonication (10% setting) for 10 min. This freeze/thaw procedure was performed three times. Afterward, the lysate was incubated for 1 h at −20 °C, followed by 15 min centrifugation at 12,000*g* at 4 °C to precipitate protein. The metabolite-containing supernatant was transferred to new tubes, dried under vacuum, prior to cleanup, while the precipitate was kept at 4 °C for protein analysis.

The dry metabolites were reconstituted in 200 μl PBS, transferred to a 96-well plate, and subject to a solid-phase microextraction (SPME) using resin-coated blades preconditioned for 30 min in methanol:water (50:50, v/v) (27). Samples were incubated with the blades for 1 h at room temperature, then the coatings were washed for 20 s in water, and the metabolites desorbed with acetonitrile:water (50:50, v/v) for 1 h. The eluate evaporated to dryness in a vacuum concentrator and sample extract reconstituted in 2% ACN and analyzed by the nLC-MS/MS system.

### Nanoflow LC-MS/MS Analysis of Metabolite Samples

Metabolite analysis was performed using a nLC-QE HF Hybrid Quadrupole-Orbitrap (Thermo Scientific) using a EasySpray microcolumn (Thermo; 2 μm, 75 μm × 50 cm) in an oven set to 40 °C. The mobile phase A was 2% ACN, and mobile phase B was to prevent ion suppression in negative mode, formic acid and TFA were eliminated from the mobile phase. The flow rate was 300 nl/min. The gradient started from 2% mobile phase B to 60% mobile phase B for 20 min and then reaching to 95% mobile phase B in 10 min and then lasted for 15 min. The Q Exactive HF mass spectrometer was operated under a dual (positive/negative) ESI switching mode in real time during data acquirement, requiring a longer gradient compared with standard analytical flow rate methods. Full mass scan (m/z 67–1000) was performed at a resolution of 60,000, with an AGC target set at 3e^6^ ions, and maximum ion injection time of 25 ms. Source ionization parameters were optimized with the spray voltage at 2.1 kV and −1.8 kV for positive and negative mode, respectively, with a transfer temperature of 300 °C. MS2 scans were performed at 15,000 resolution, with a maximum injection time of 64 ms, using NCE steps of 10, 20, and 40. The dynamic exclusion was set to 10 s.

### Metabolomics Data Analysis

Raw data files were converted to mzML format and split into positive and negative spectra using msConvert prior to analysis using MS-DIAL4 (28). “Linear-weighted moving average” was used for peak detection, with a minimum peak height set to 20,000. Afterward, spectral centroiding was performed by integrating the mass spectrum across a ±0.01 and ±0.025 Da range in MS1 and MS2 respectively. Spectra were searched against MS-DIAL metabolomic or lipidomic MSP library with tolerance of 0.025 and 0.05 for MS1 and MS2, respectively. Common adducts ([M + H]+, [M+NH4]+, [M+Na]+, [M-H]−, [M-H2O-H]−, [M+Cl]−, etc.) were annotated prior to identification. To assess the FDR, implausible adducts (*e.g.*, [M+Be]+/−) were defined as decoys during the database search along with native adducts. A stringent confidence cutoff score (>50) was used to improve the confidence of annotated metabolite matches using an empirically determined FDR of <0.1 probability (relative to decoy matches). Quality control data (QC) were specified as a reference file for alignment between samples. Data matrices were exported as tab delimited text files for subsequent enrichment analysis.

### Data Analysis and Pathway Enrichment

Bioinformatic analysis was performed using R: *A language and environment for Statistical Computing* (R Foundation for Statistical Computing, http://www.R-project.org) using an in-house pipeline (29). Briefly, the table of feature intensities was log transformed and quantile normalized. Untargeted metabolomics enrichment analysis was performed by MetaboAnalyst (15, 30). The LIMMA (31) R package was used for differential protein and metabolite analysis to generate ranked lists, after Benjamini–Hochberg FDR correction, for subsequent pathway (gene set) enrichment analysis using the fgsea R package (32) with statistical significance calculated using 10,000 permutations. For the joint protein and phosphosite feature matrixes, a combined ranked list was generated where in the case of duplicate gene names, the entry with the greatest absolute rank value was retained. The Cytoscape EnrichmentMap module (33) was used to visualize the respective enrichment results in a network layout after clustering the pathways based on feature overlap. Boxplots were generated with the ggplot R package, with the centerline representing the median, upper, and lower hinges representing the 75th and 25th percentiles, respectively, and with whiskers extending from the hinges to the most extreme values no further than 1.5 ∗ IQR (interquartile range) from the hinge. Data extending beyond the end of the whiskers were plotted individually.

### Protein–Protein Interaction and Metabolic Model Database Parsing

Metabolic models were downloaded from MetaboAnalyst (www.metaboanalyst.ca). Where available, E.C. codes were converted into gene/protein names based on mappings from Uniprot (34). For each pathway, a base gene set was derived based on these genes alone. To expand the gene sets and increase the number of components associated with metabolic pathways, we cross-mapped curated PPIs from InWeb_IM (22) (human) and iRefIndex (35) (other species) using two approaches: “*Expanded*” gene sets incorporated all first-degree interactors, while “*Neighborhood*” gene sets were based on the most confident first- and second-degree interactors to reach a target gene set size (250) based on scoring from the PPI databases. Similarly sized control gene sets were also created by expanding with (1) the same number of random proteins drawn from the PPI network, or (2) the same number of random gene products from the PPI network that were second-degree neighbors to each other (random subnetworks). “*Base*” gene sets consisting of enzymes alone were also generated directly from the metabolic models for comparison.

### Visualization of Pathway Protein–Metabolite Subnetworks

For visualizing pathway-based protein–metabolite subnetworks, network files were generated incorporating PPIs and metabolite reaction pathways. Since reaction information was not included with the metabolic models downloaded from MetaboAnalyst, specific pathway information was retrieved from MetScape (human). Files were integrated manually to visualize interaction networks encompassing metabolites, reactions, enzymes, and associated proteins (human gene products). Then, for a specified pathway, the *Neighborhood* protein interaction subnetwork was added using a script after having been previously parsed at the database integration step. Subnetworks were visualized with Cytoscape. Pathway nodes with only one interactor (*e.g.*, nodes that mediated no interconnection) that were not detected in any dataset were removed to declutter the final image. Nodes were colored based on changes between conditions if they were detected, while shapes were specified to distinguish metabolites, reactions, genes, and PPI associated proteins, as described in figure legends.

## Results

### Integrating Metabolic Models With Protein–Protein Interactions to Define Pathway-Centric Subnetworks

Metabolic models based on curated pathway annotations, such as KEGG (36), offer a valuable but incomplete mapping of enzymatic reactions relative to the rest of the proteome. For example, enzymes in the human MFN metabolic model (curated aggregation of BiGG, KEGG, and the Edinburgh model) map to only 1475 unique genes out of the ∼23,000 open reading frames encoded by the human genome (supplemental Table S1), which constrains traditional gene set enrichment analyses. Since perturbations in metabolic pathways caused by physiological regulation or disease likely propagate through a wider network encompassing proteins that interact physically and functionally with these enzymes (37, 38), we reasoned that high-confidence PPI could be used to extend the coverage of metabolic-pathway models to a greater fraction of the proteome.

**Fig. 1:**
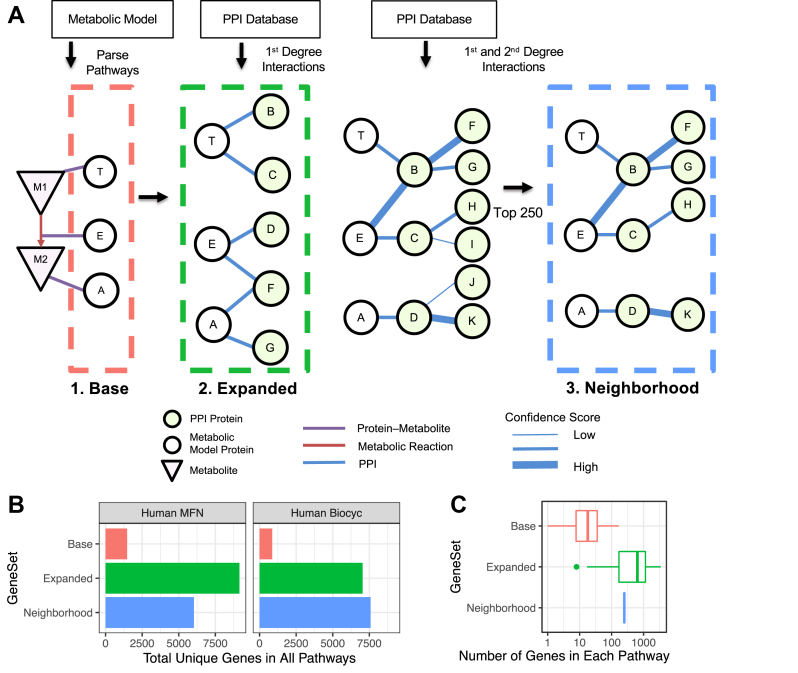
**Integrative metabolomic and proteomic analysis using MOMENTA.***A*, metabolic models and protein–protein interaction (PPI) networks are integrated to define metabolic pathway subnetworks (*Base*, *Expanded* or *Neighborhood*) and to derive enhanced gene sets. *B*, plot showing the number of total unique genes across all pathways in the Human MFN and Human Biocyc metabolic models for the *Base*, *Expanded*, and *Neighborhood* gene sets. *C*, distribution of pathway sizes (number of genes) for the *Base*, *Expanded*, and *Neighborhood* gene sets derived from the Human MFN metabolic model. MOMENTA, multiomic metabolic enrichment network analysis.

To examine this systematically, we started with metabolic models supported by MetaboAnalyst (15), a leading metabolomic analysis platform and aggregator of curated metabolic pathway models with plans to extend support to more metabolic models in the future. We extended the metabolic model pathways with scored binary interactions, downloaded from the InWeb_Im (22) and iRefIndex (35) databases, based on compiling either: (i) all the direct (first-degree) interaction partners (“*Expanded*”) of annotated enzymes (Fig. 1*A*), which markedly increased the number of associated gene products (from 1475 to 9145 for MFN; Fig. 1*B*), but created a range of gene set sizes (supplemental Table S2 and Fig. 1*C*) that impacts enrichment statistics (39); or (ii) both the direct (first-degree) and nearest neighbor (second-degree) interaction partners of enzymes, followed by pruning each subnetwork to generate a uniform and optimal (39) gene set size (“*Neighborhood*”) (Fig. 1*A*), which substantially increases the number of unique genes/proteins for all pathway models (Fig. 1*B*) while creating a uniform distribution of gene set sizes for standardized enrichment (statistical) scoring (Fig. 1*C*). Extending the metabolic pathways through the PPI network, as opposed to metabolic network, maintains the metabolic pathway at the geometric center of the subnetworks as much as possible, which supports the intended use of proteomic data to study metabolic pathways.

Our method of network integration results in these *Expanded* and *Neighborhood* gene set definitions, which we can apply in a boosted enrichment analysis pipeline to examine differences in metabolic pathways starting from experimental proteomic profiling data. Benchmarks for the obtained enrichment results include both standard “*Base*” gene sets, representing traditional annotated metabolic pathways (*i.e.*, enzyme alone gene sets), and against two control gene sets, based on globally randomized sets of proteins (“*Control 1*”) or randomized interaction subnetworks (“*Control 2*”).

### Metabolic Pathway Changes in Breast Cancer Cells in Response to Glucose Starvation

**Fig. 2:**
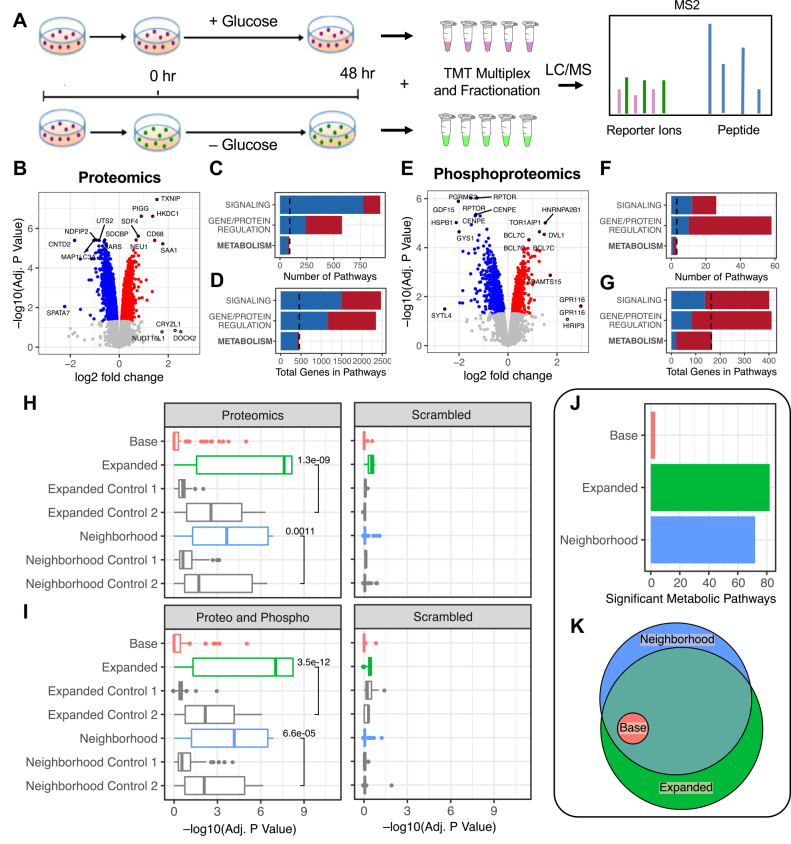
**MOMENTA applied to a glucose-starved *in vitro* breast cancer cell line model.***A*, MDA-MB-231 cells, cultured in standard media or glucose-starved media, were subjected to TMT-multiplexing and LC/MS-based proteomic and phosphoproteomic analysis. *B*, differential analysis of the proteomic data showing top differential proteins. *C*, number of pathways enriched in each of three major biological categories from the proteomic analysis. *D*, total number of unique genes mapping to pathways based on major biological categories. *E*, differential analysis of phosphoproteomic data showing top differential phosphosites across all conditions. *F*, number of pathways enriched in major biological categories for the phosphoproteomic analysis. *G*, total number of unique genes mapping to pathways based on the phosphoproteomic enrichment analysis. *H*, boxplot of individual pathway enrichment significance for all metabolic pathways in the Human MFN model using the proteomic data alone for the *Base* (enzyme only), *Expanded*, and *Neighborhood* gene sets. Matched control gene sets with the same number of PPI components added at random (Control 1) or from an alternative area in the PPI network (all second-degree neighbors, Control 2). The same enrichment analysis on scrambled proteomic data, after gene annotations were randomized. *I*, plot of global pathway significance across gene sets as in *A*, with the enrichments based on the combined proteomic and phosphoproteomic data. *J*, number of significant metabolic pathways (*p* < 0.05) enriched in the phospho/proteomic profiles using the *Base*, *Expanded*, and *Neighborhood* gene sets. *K*, overlap of significant metabolic pathways (Adj. *p* Value < 0.05) enriched in the phospho/proteomic profiles using the *Base*, *Expanded*, and *Neighborhood* gene sets. MOMENTA, multiomic metabolic enrichment network analysis; PPI, protein–protein interaction; TMT, tandem mass tag.

As a demonstration of utility, we first applied MOMENTA to examine metabolic constraint and pathway adaptation in a cancer cell line model system (supplemental Fig. S1). Tumors are known to exhibit metabolic stress stemming from competitive growth and reduced angiogenic perfusion yet are resilient (40, 41). As a model, we cultured MDA-MB-231 triple-negative breast cancer cells in both standard and glucose-free media for 48 h to mimic nutrient deprivation (Fig. 2*A*). For replicate cultures, we measured proteins and phosphopeptides by isobaric tandem mass tag (TMT) multiplexing and nLC/MS, quantifying 7214 proteins (supplemental Table S3*A*) and 5785 phosphosites (supplemental Table S4*A*), respectively after normalization (see supplemental Tables S3*B* and S4*B* for unnormalized data with identification annotation). Differential analysis identified 3055 high-confidence (Adj. *p* Value < 0.05) proteins and 1528 phosphosites.

The proteomic analysis showed consistent differential expression (Fig. 2*B* and supplemental Fig. S2*A*), including upregulation of TXNIP, a downstream target of IGF1 (insulin) signaling that is linked to cellular redox signaling (42). We subjected the ranked differential expression profiles to a standard, broad GSEA to examine functional patterns. Conventional (GSEA) analysis using a large library of curated gene sets (33) revealed metabolic stress consistent with glucose starvation (supplemental Fig. S2*B* and supplemental Table S5); however, the returned metabolic pathways were broadly defined, represented only a small minority of the major biological themes deemed significant (Fig. 2*C*) and encompassed only a small fraction of the number of total gene products as compared with other major functional categories (Fig. 2*D*).

Differential analysis of the phosphoproteomic data likewise showed a robust signature (Fig. 2*E* and supplemental Fig. S2*C*), including downregulation of the metabolism master regulator, RPTOR, consistent with reduced nutrient levels (43), and additional orthogonal signaling pathways reflective of global changes in intracellular kinase activity (supplemental Fig. S3). Again, however, only high-level metabolic terms (Nitrogen, Lipid and Carbon utilization) were returned (supplemental Fig. S2*D* and supplemental Table S6) in the general-purpose GSEA, whereas more fine-grained metabolic pathways were underrepresented in the enriched terms (Fig. 2*F*) and mapped to a sparse subset of the differential phosphoproteins deemed differential in the data (Fig. 2*G*).

In order to more thoroughly interrogate metabolic signatures based on the proteomic data, we performed GSEA using the MOMENTA-derived gene sets. In this analysis of the proteomic and phosphoproteomic data, we compared the *Base* enrichment results, representing existing methods, with our computationally derived *Expanded* and *Neighborhood* gene sets. Further, we compared against the *Control* gene sets to test whether or not observed difference was better than would be expected from random chance. To first assess the performance of the enrichment results at a global level, we examined the global significance distribution across all pathways. Strikingly, for the proteomic data alone, using the MFN and Biocyc-derived metabolic models, the *Expanded* and *Neighborhood* gene set analyses produced markedly increased metabolic pathway enrichment significance results relative to either the *Base* or *Control* gene sets (Fig. 2*H* and supplemental Fig. S4*A*). To compare global enrichment results, we primarily use these distributions of pathway significance to show effects on the statistical tests across all pathways. This effect was eliminated when the underlying protein and phosphoprotein data matrixes were randomized, effectively removing the signature derived from the curated molecular interaction network (Fig. 2*H* and supplemental Fig. S4*A*), demonstrating that the signal is due to underlying biologically functional connections between proteins.

An even more pronounced effect was observed after subjecting the combined proteomic and phosphoproteomic data to enrichment with MOMENTA (Fig. 2*I* and supplemental Fig. S4*B*), which provided increased gene and pathway coverage over the proteomic data alone. Using the MFN model, the number of significant (Adj. *p* Value < 0.05) pathways increased 25-fold, from only three with the *Base* gene sets to 82 and 72 in the *Expanded* and *Neighborhood* subnetworks, respectively (Fig. 2*J* and supplemental Tables S7–S9); all three of the significant pathways in the *Base* analysis were also captured in the *Expanded* and *Neighborhood* results (Fig. 2*K*). While the majority of significant pathways for *Expanded* and *Neighborhood* are shared, we can examine the individual pathway results to explain differences. For example, Purine Metabolism is significant in the *Expanded* results (Adj. *p* Value 0.0009; supplemental Table S8) but not the *Neighborhood* results (Adj. *p* Value 0.8). In this case, when we examine the gene set sizes, we see that the Purine Metabolism pathway comprises 3449 genes in the *Expanded* case compared with 250 in *Neighborhood* (supplemental Table S2). These differing results are consistent with nonspecific enrichment observed in GSEA analyses when very large gene sets are used and why they are often excluded. However, comparison to an orthogonal method is necessary to ultimately validate results.

In order to control for overlapping pathways (genes in multiple pathways) and visualize the overall enrichment results, we used the Enrichment Map Cytoscape plugin to graph the metabolic pathway terms returned by the *Base*, *Expanded*, and *Neighborhood*, which clustered significant gene sets into biological modules based on shared protein/gene annotations. As shown in supplemental Fig. S5, MOMENTA documented a profound decrease in global metabolic pathway activity in the MDA-MB-231 cells following glucose-withdrawal, consistent with lower energy consumption and holistic pathway remodeling resulting from the withdrawal of a primary carbon source. Major themes included a predominant downregulation of pathways related to sugar metabolism, lipid synthesis, as well as upregulation of adaptive pathways, such as Urea Cycle/Amino Group and Lysine metabolism (supplemental Fig. S5), consistent with the utilization of amino acids as alternative fuel source by cancer cells (1).

### Metabolomic Profiling of Glucose-Starved Cancer Cells Consistent with MOMENTA Projections

**Fig. 3:**
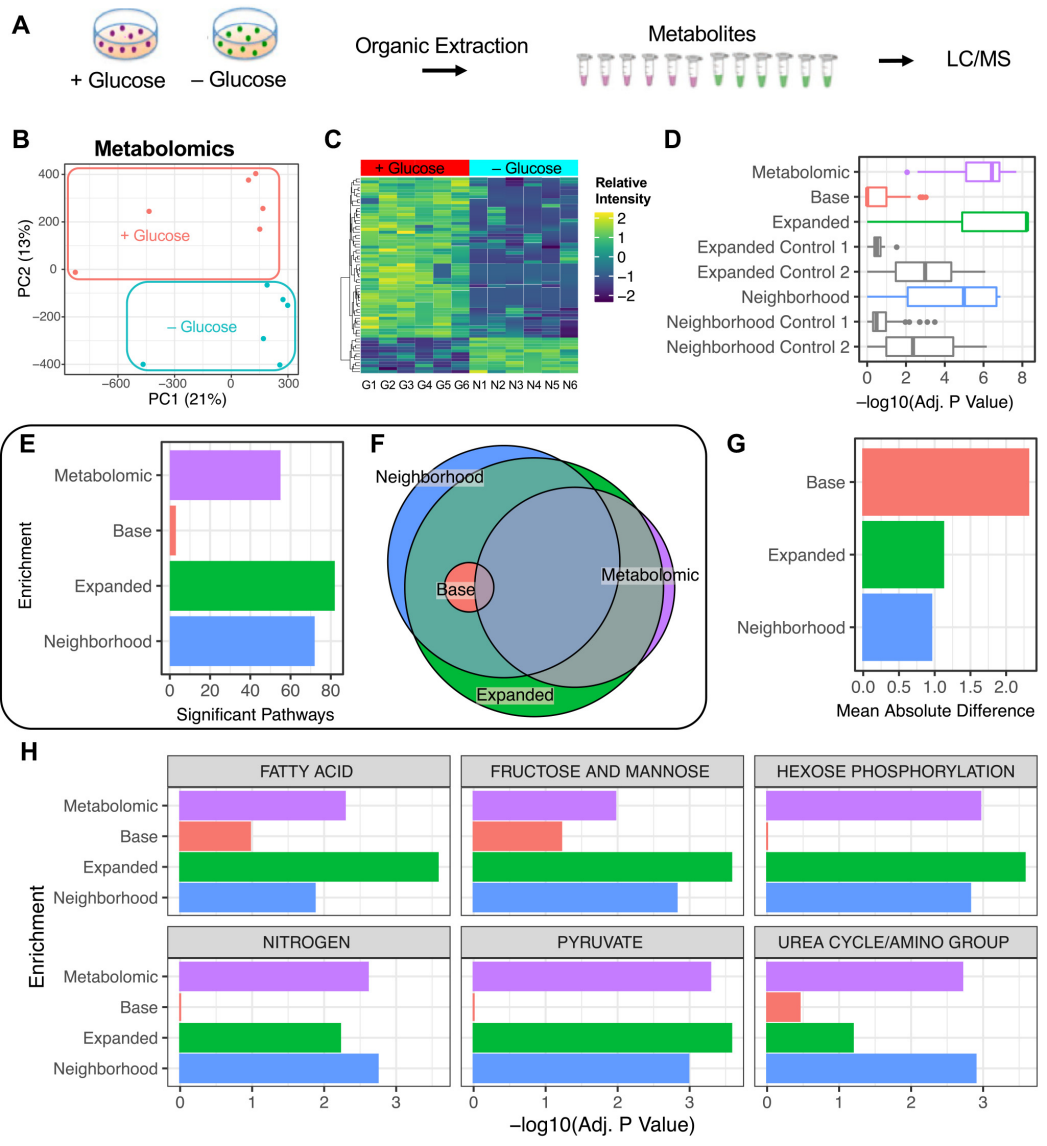
**Experimental validation of metabolomic changes in breast cancer cell line.***A*, experimental workflow of metabolomic profiling of glucose-starved cell line. *B*, PCA dimensionality reduction of unbiased metabolomic profiles showing partitioning of Glucose *versus* No Glucose samples. *C*, heatmap of top differential annotated metabolites identified using a decoy adduct strategy. *D*, global pathway enrichment results comparing the global significance of phospho/proteomic pathways with metabolomics-based analysis using the same *Base*, *Expanded*, *Neighborhood*, and *Control* gene sets. *E*, number of significant metabolic pathways (Adj. *p* Value < 0.05) enriched in the metabolomic and phospho/proteomic data using the *Base*, *Expanded*, and *Neighborhood* gene sets. *F*, overlap of significant (Adj. *p* Value < 0.05) metabolic pathways enriched in the metabolomic data and phospho/proteomic data using the *Base*, *Expanded*, and *Neighborhood* gene sets. *G*, mean absolute difference of pathway significance between the Metabolomic and phospho/proteomic enrichment results. *H*, significance of select metabolic pathways across the metabolomic and phospho/proteomic profiles. PCA, principal component analysis.

The use of computationally randomized controls and data goes a long way in establishing confidence in the enrichment analysis. However, ultimately biological validation requires testing results using orthogonal methods. In order to validate the results from the MOMENTA metabolic pathway interrogation, we performed global metabolomic profiling on the same glucose-starved cancer cell samples in parallel with our phospho/proteomic analysis (Fig. 3*A*). Global analysis of untargeted metabolomics showed samples clustering based on glucose withdrawal (Fig. 3*B*) and using an empirically controlled (decoy adduct) metabolite identification pipeline (44), we found a robust signature from replicate sample measurements across both conditions (Fig. 3*C*).

To systematically compare the metabolomic pathway enrichment results produced by MOMENTA and direct experimental detection of differential metabolites, we integrated the MetaboAnalyst metabolic enrichment results based on the metabolomic data with the phospho/proteomic enrichment analysis using the same gene set (pathway) definitions (supplemental Table S10). Notably, the metabolomic profile enrichment results were in closest agreement in terms of metabolic pathway significance with the *Neighborhood* and *Expanded* subnetworks, followed distantly by *Base* and *Control* gene sets (Fig. 3*D*). We are able to compare these distributions between the metabolomic data and the proteomic data because in both cases, enrichments are based on the same list of pathways, derived from the metabolic models, as described previously. Additionally, the *Expanded* and *Neighborhood* enrichment results produced a comparable number and significantly overlapping (Adj. *p* Value < 0.05) of pathways as reported by the Metabolomic experiments, relative to the sparse *Base* enrichments (Fig. 3*E*). Consistent with a high degree of agreement, the significant pathways found with the *Expanded* and *Neighborhood* subnetworks overlapped with the majority of the enriched pathways detected by direct metabolomic profiling (Fig. 3*F*); the mean absolute difference in significance returned for each pathway was greatest between the *Base* and Metabolomic enrichments, reflecting the least agreement (Fig. 3*G*). While there is a great deal of overlap between the proteomic and metabolomic analysis, examination of individual pathway results reveals technical reasons for cases where there is not agreement. Vitamin B12 metabolism is significantly altered in the metabolomic enrichment results, however, not in any of the proteomic results. Examination of the gene set composition for this pathway reveals that in this metabolic model it is only annotated with two genes (supplemental Table S2), resulting in a very small *Expanded* gene set that is unlikely to produce a robust signal (33). Similarly, among the set of pathways significantly enriched in the proteomic data but not that metabolomic data are a number of lipid and fatty acid–related pathways (*e.g.*, Glycosphingolipid biosynthesis, fatty acid oxidation) composed of metabolite molecules that are unlikely to be detected in our metabolite profiling method. Despite picking a method to be as global as possible with metabolite measurements, this illustrates the impact metabolite chemical diversity has on the feasibility of comprehensive metabolite profiling.

Notably, looking at representative pathways, while the *Base* gene set analysis showed some signal in relevant pathways such as Fructose and Mannose Metabolism, it produced hardly any signal in Hexose Phosphorylation, Nitrogen, or Pyruvate Metabolism, all of which were deemed significantly altered in the direct metabolomic survey, as well as the *Expanded* and *Neighborhood* analyses (Fig. 3*H*). Consistent with a wider range of gene set sizes (Fig. 1*C*), the *Expanded* correlation with the metabolic results was less pronounced than for the *Neighborhood* approach, suggesting that gene set size distribution contributes some degree of noise, as expected, with uniform subnetwork sizes producing more robust findings.

### MOMENTA Reveals Metabolic Variation Across Cancer Cell Types

**Fig. 4:**
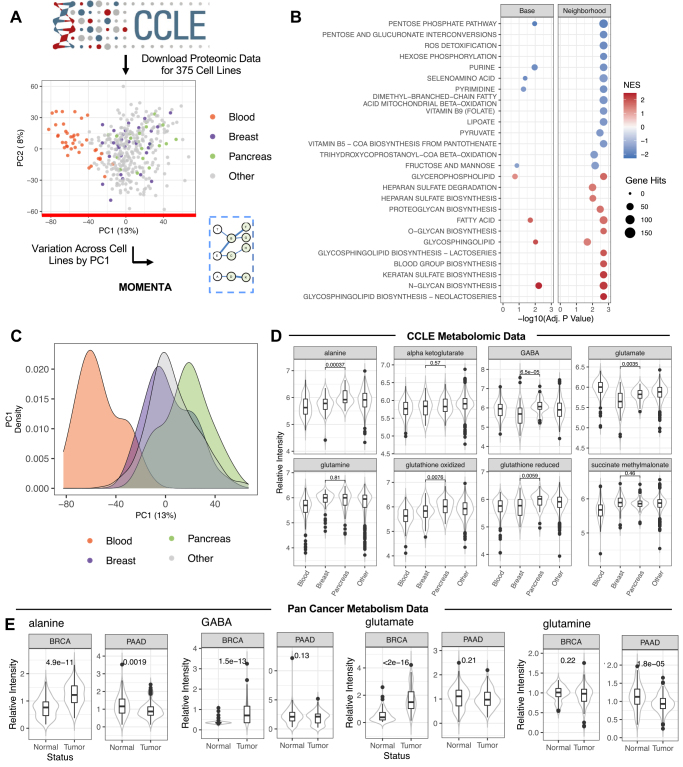
**MOMENTA analysis of metabolic variation across diverse cancer cell lines.***A*, workflow for CCLE proteomic profiling data processing and MOMENTA analysis. *B*, enrichment results for top *Neighborhood* pathways (Adj. *p* Value < 0.01) for enrichment based on global profile PC1. *C*, distribution of tissue derived cancer cell lines across PC1. *D*, relative intensity of glutamate metabolism pathway metabolites in CCLE for highlighted cancer cell types, with *p* Values for comparison between Breast and Pancreas based on Kruskal–Wallis test. *E*, relative intensity of metabolites in tumor and normal tissue samples for both Breast (BRCA) and Pancreas (PAAD) cancers showing corresponding *p* Values (*t* test). CCLE, Cancer Cell Line Encyclopedia; MOMENTA, multiomic metabolic enrichment network analysis.

A recent in-depth proteomic profiling of hundreds of cancer cell lines (45) offers an important resource to study variation in cancer cell pathways and, ultimately, better understand unique susceptibilities to targeted therapeutics based on molecular phenotypes. Dimensionality reduction with principal component analysis (PCA) of the quantitative proteomic profiles for 375 cell lines, representing a diversity of tissues of origin (*e.g.*, Breast, Pancreas), revealed broad variation across the large number of blood and solid tumor cell types surveyed (Fig. 4*A*), confirming the original report, which mentions metabolic pathways in a cursory discussion of broad pathways (*e.g.*, glycolysis and nucleotide metabolism).

In order to determine the extent of variation in metabolic activity across the cancer cell types, we performed a more extensive MOMENTA *Neighborhood* enrichment analysis based on the protein (gene) rankings according to their loadings on the first principal component (PC1) (Fig. 4, *A* and *B* and supplemental Table S11). The top most significantly (Adj. *p* Value < 0.01) varying pathways from the *Neighborhood* subnetwork model mapped to over 20 pathways related to the metabolism of extracellular glycans, diverse lipids, and oxidation related processes, among others (Fig. 4*B*), which have previously been implicated in cancer signaling (46). These pathways represent systems that are differentially regulated across the profiled cancer cell types.

Visual inspection of the tumor line distributions along PC1 revealed a strong separation of those derived from blood-borne cancers from those originating from solid tumors (Fig. 4*C*), consistent with the original analysis. This also is consistent with the findings of Heparan Sulfate and Glycan pathways, which are expected to vary substantially between blood and solid organ cell types. To confirm these patterns, we downloaded and clustered the metabolomic data available for 225 metabolites in 928 cell lines (47) and found a similar stratification of cancer cell types (Blood, Breast, Pancreas, Other) along PC1 in both our metabolomic and proteomic PCA analyses (supplemental Fig. S6). We then cross-mapped all metabolites to the corresponding pathways implicated by our MOMENTA *Neighborhood* analysis (Fig. 4*B*) and found 39 (out of 43 detected) were significantly altered across the subtypes used for comparison (Blood, Breast, Pancreas, Other; ANOVA, *p* < 0.05) providing validation of variation across cell types in our top pathways (supplemental Fig. S7). Further, when we looked at particular metabolites from these same significant pathways in clinical metabolomic data recently reported for Breast and Pancreas cancers (9), we found 11 metabolites (out of 14 detected) were significantly altered (*t* test, *p* < 0.05) between the tumor and adjacent normal tissue (supplemental Fig. S8), but not concordantly across the two tissues of origin (two metabolites were significantly concordantly regulated, one was not significantly regulated).

While some separation was evident among certain solid organ tissues, such as Breast and Pancreas, based on PC1, more in-depth molecular phenotyping was deemed warranted to define significant metabolic. To focus on the specific differences between these two cell types with clinical data (Breast and Pancreas), we repeated the MOMENTA *Neighborhood*-based enrichment analysis to compare the Breast and Pancreas cell lines and found 23 significantly (Adj. *p* Value < 0.05) differential metabolic pathways (supplemental Fig. S9). Again, we were able to independently verify these findings with clinical metabolite measurements from the Pan Cancer Metabolism Data, of the ten metabolites mapping to these same 23 pathways, we found seven were significantly differentially regulated in Breast and Pancreas tumors as compared with normal tissue (supplemental Fig. S10). Discordant directionality in this case may be indicative of metabolite substrates and intermediates being depleted by upregulation of protein regulators (*e.g.*, phosphorylation events) in certain pathways. One notable pathway predicted to show differential activity mapped to Glutamate metabolism, where the associated metabolites alanine, GABA, glutamate, and glutathione were found to significantly different between both breast and pancreatic cancer–derived cell lines, which show metabolite abundances in the cell lines relative to one another (Fig. 4*D*), and between tumors and normal adjacent tissue primarily in Breast relative to Pancreas cancers (Fig. 4*E*), though note these data do not adjust for differing normal levels of metabolite abundance in different tissues. This is potentially clinically significant since recent research has pointed to these metabolites as potential cotargets for immuneoncology therapeutics (48). Our analysis suggests that metabolomic phenotyping may be an important biomarker to stratify cancers for susceptibility to this therapeutic strategy.

### Evaluation of the Proteomic Profiles of Clinical Samples Using MOMENTA

**Fig. 5:**
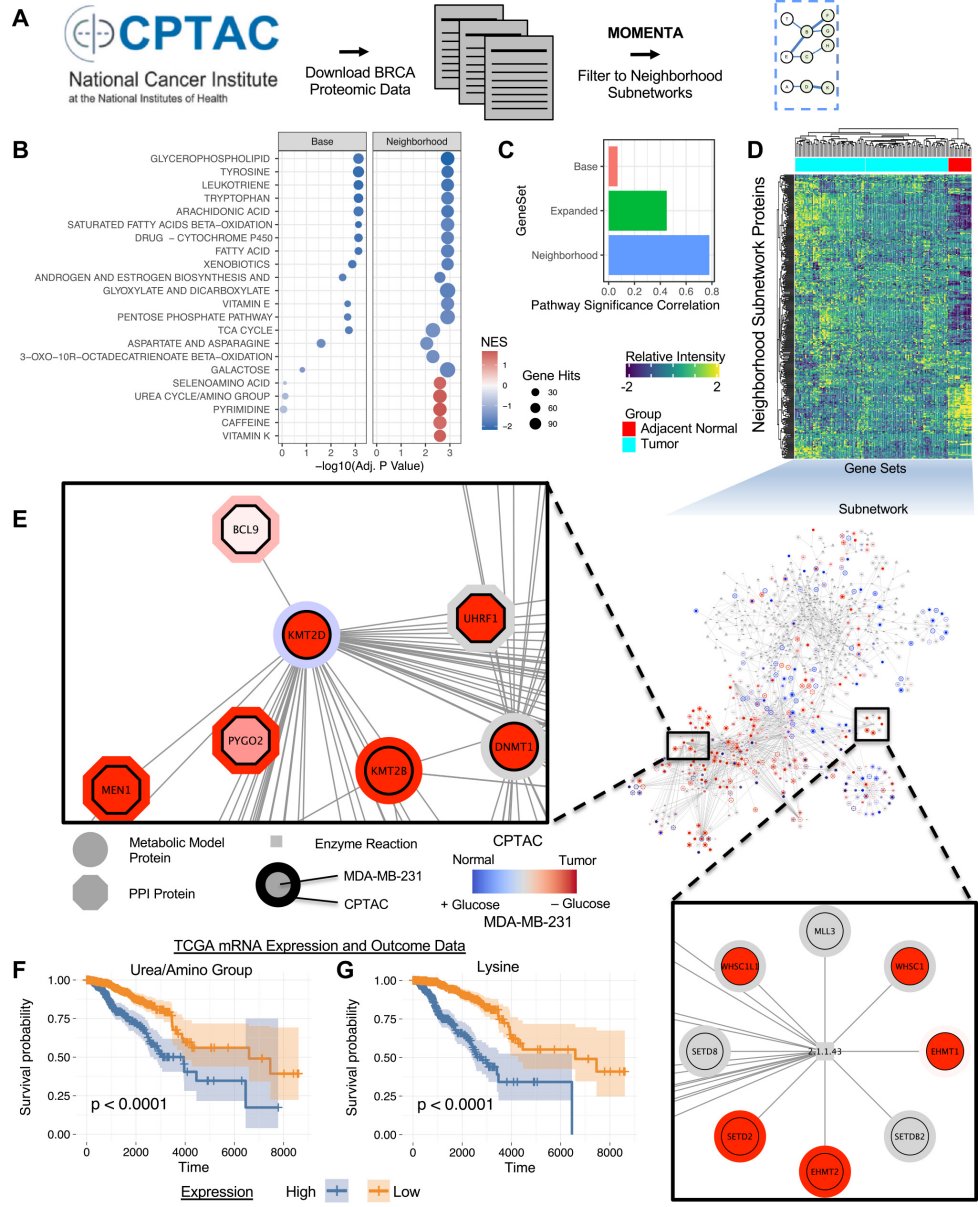
**Analysis of CPTAC clinical proteomics profiles using MOMENTA-based *Neighborhood* gene sets.***A*, analysis scheme for breast cancer data downloaded from the CPTAC database. *B*, enrichment results based on *Neighborhood* gene sets comparing significant (Adj. *p* Value < 0.01) pathways in tumors and adjacent normal samples. *C*, correlation of pathways inferred using different gene sets between the *in vivo* CPTAC and *in vitro* MDA-MB-231 cell culture data. *D*, heatmap of CPTAC proteomic results corresponding to the *Neighborhood* Urea Cycle/Amino Group and Lysine pathways used for hierarchical clustering of tumor and adjacent normal samples. *E*, molecular network of combined Urea Cycle/Amino Group and Lysine metabolic pathways and their associated *Neighborhood* proteins. Nodes are colored to show CPTAC and MDA-MB-231 expression patterns. *F*, Kaplan–Meier curve showing clinical outcomes based on TCGA-BRCA mRNA expression data reported for select proteins in the Urea Cycle/Amino Group *Neighborhood*. *G*, Kaplan–Meier curve for outcomes based on TCGA-BRCA mRNA expression data for select proteins from the Lysine *Neighborhood*. CPTAC, Clinical Proteomic Tumor Analysis Consortium; MOMENTA, multiomic metabolic enrichment network analysis.

Having demonstrated the utility of the MOMENTA gene set enrichment to more thoroughly interrogate differences in the metabolic activity of cell lines, we extended our studies to analyze the proteomic profiles generated for patient-derived tumor specimens as reported in a recent CPTAC (Clinical Proteomic Tumor Analysis Consortium) breast cancer study (Fig. 5*A*) (49). Consistent with previous research on tumor metabolic suppression and adaptation (9), our MOMENTA-based *Neighborhood* enrichment analysis revealed a global downregulation in many metabolic pathways (Fig. 5*B* and supplemental Table S12) and an increase in specific metabolic pathways, such as Urea Cycle/Amino Group, Lysine, and Pyrimidine metabolism (Adj. *p* Value < 0.01). When compared with the nutrient stressed cancer cells studied previously, we see strong agreement in the metabolic pathway perturbations in the *Neighborhood* enrichment results (Fig. 5*C*) and focused on the Urea Cycle/Amino Group together with the Lysine pathway, due to overlapping features (supplemental Fig. S5), for further interrogation.

To independently confirm the pathway composition and definitions for the metabolic models used in the MOMENTA analysis, we downloaded tumor metabolite measurements from the Pan Cancer Metabolism Data project (9). We plotted the compound ion intensities reported for quantified metabolites in the Lysine and Urea Cycle/Amino Group metabolic pathways in different tumors (supplemental Fig. S11). Strikingly, of 14 cross-mapped metabolites, 13 (93%) were found to be significantly (Adj. *p* Value < 0.05) increased in breast cancer as compared with adjacent normal tissue. Moreover, among all cancer types, 11 of 18 metabolites associated with Lysine and Urea Cycle/Amino Group metabolism exhibited significantly (Adj. *p* Value < 0.05) altered levels in tumors (supplemental Fig. S12), though these data may be noisier due to comparisons across multiple cancer types.

Approximately half (130 of 266) of the proteins in the *Neighborhood* subnetworks were significantly (Adj. *p* Value < 0.05) differentially altered, showing substantial biochemical rewiring of these metabolic pathways and functionally associated proteins. While metabolic rewiring in nitrogen utilization toward pyrimidine synthesis is a well-established biochemical signature of cancer (9), this external validation serves as important confirmation that specific pathway metabolites predicted by MOMENTA solely on proteomics data are indeed significantly altered. Unsupervised hierarchical clustering based on just the levels of enzymes and associated proteins implicated by the MOMENTA *Neighborhood* analyses that map to Urea Cycle/Amino Group and Lysine pathways also readily discriminated between the tumor and adjacent normal samples (Fig. 5*D*).

We extended our analysis to examine the physical associations underlying each differential subnetwork, which revealed mechanistic relationships between metabolites, enzymes, and interacting (PPI) proteins contributing to the enrichment signature (Fig. 5*E*). To investigate the functional basis of the Urea/Amino and Lysine pathway groupings, we overlaid the CPTAC tumor protein expression enrichment results together with the results of our *in vitro* MDA-MD-231 cell line analysis. Highly connected components in the combined subnetwork (Fig. 5*E*) included the Histone-lysine N-methyltransferase KMT2D, which was elevated in both datasets, suggesting metabolic modeling is associated with epigenetic feedback. KMT2D has been reported to physically interact with multiple proteins associated with cancer and tumor progression, including BCL9L (50), MEN1 (51), and UHRF1 (52). Related enzymes, and their interacting (PPI) protein neighbors, were likewise strongly upregulated in the phospho/proteomic data, including SETD2, another histone lysine methyltransferase implicated as a tumor suppressor (53), suggestive of a metabolic regulatory mechanism operating in nutrient-deprived breast tumors.

To independently validate the significance of these findings using orthogonal data, we downloaded the breast cancer mRNA expression and associated clinical outcomes data (TCGA-BRCA) from the cBioPortal. After data processing, we explored the mRNA expression profiles of 1045 patients to test prognosis significance of our MOMENTA *Neighborhood*-based signatures after applying a LASSO Cox model to automatically perform feature (gene) selection. We then classified the patients into high-risk and the low-risk groups based on a subset of the Urea Cycle/Amino Group and Lysine gene features. Finally, we generated a Kaplan–Meier plot to examine the survival difference between the high-risk and low-risk groups and found high expression of a subset of genes in both the Urea Cycle/Amino Group (Fig. 5*F*) and Lysine (Fig. 5*G*) *Neighborhood* subnetworks were significantly (*p* Value < 0.0001) associated with decreased overall survival probability. Taken together, these results establish the ability of MOMENTA to infer reproducible and clinical meaningful changes in cancer cell metabolic activity that are associated with disease progression.

## Discussion

By leveraging existing knowledge, MOMENTA can provide unexpected mechanistic insights into complex biological processes associated with cellular metabolism. While recent progress continues to be made on correlative relationships between the metabolome and genome (54), we have demonstrated the utility and importance of expanded functional models for exploring connections between metabolism and the proteome. We demonstrated the utility of our approach for leveraging widely available proteomic data to study more precisely the metabolic pathway perturbations that occur in tumor cell lines and then extended the analysis to study metabolomic variation in tumor samples. While we established compelling agreement between direct metabolite measurements and the metabolic pathway signatures identified from proteomic profiles, the experimental metabolite coverage reported to date is often so limited that comprehensive and statistically significant interrogation of metabolic pathways is not possible using metabolomic data alone. Hence, MOMENTA addresses an important existing unmet research need. While we demonstrate a greater degree of signal in global metabolic pathway enrichments based on proteomic measurements, over existing methods and randomized controls, it is important to remember that the goal of identifying a small number of actionable mechanisms in many contexts of biological research is not necessarily served by this metric. Boosting signal is helpful in cases where existing methods do not reveal any signatures, or reveal too few, but corroborating analyses will be required to help narrow down and validate pathways.

One limitation of this approach is the reliance on existing metabolic pathway annotations, which are prone to error, incompleteness, and bias. While other methods capable of network inference and the identification of *de novo* pathways have been reported (25, 55), these methods may still be impacted by the accuracy of individual molecular interactions and pathways are useful to help narrow the search space for validation. Additionally, a limitation of this method is the underlying source networks. While the PPI are confidence-weighted, this method does not capture or model the diverse functional relationships between diverse biomolecules. Both protein and small molecule cofactors have tremendously impactful roles on regulating metabolic activities, and these functional relationships are not well modeled in this approach, beyond their inclusion as network nodes if annotated in the metabolic model. As the reliability of PPI and metabolic models improves, our analysis framework can be easily updated to incorporate new, and increasingly accurate, metabolic pathway and interactome information. Ultimately, new methods of network distillation may be needed to model complex biomolecular functions while still maintaining an ability to perform rapid, interpretable, and global analysis.

An additional caveat is that certain aspects of metabolic regulation that depend on localization to specific subcellular compartments may not be captured (23) by any methods based on global proteomic or metabolomic measurements. However, by incorporating more proteins into the network-based analysis that may be components of biomolecular complexes involved in spatial compartmentalization, we provide a path to incorporate more granular functional information or, ultimately, sample-specific PPI data into the enrichment analysis. Ultimately, defining a detailed molecular mechanism will require follow-up and validation with more specialized, targeted methods.

To stimulate broad community access to this rapid exploratory metabolic pathway analysis, we provide precomputed metabolic gene sets (github.com/cnsb-boston/MOMENTA) for integration into existing enrichment workflows, as well as a stand-alone R script to enable automated enrichment analysis from MOMENTA inferred subnetworks, along with efficient visualization of the combined results to examine metabolic pathway enrichment in one or more datasets. Since high-throughput identification of metabolites remains challenging (56), future efforts to understand the regulation and roles of metabolic pathways in cancer and other diseases will benefit from improved integration models incorporating additional functional relationships and data types.

## Data Availability

Data for this project have been deposited to the MassIVE archive (accession code MSV000084811; https://doi.org/10.25345/C57X00). We also uploaded our proteomic data to MS-Viewer (search key zsl8gjg385 and fmmj3pysq4). Code for our in-house analysis scripts for proteomic data normalization, processing, plotting, and pathway enrichment is available on github (github.com/cnsb-boston/Omics_Notebook). Gene sets and pathway network files have also been uploaded to github and are freely available (github.com/cnsb-boston/MOMENTA).

## Supplemental data

This article contains supplemental data.

## Conflict of interest

The authors declare no competing interests.
