# Supplementary material for: Multiomic Metabolic Enrichment Network Analysis Reveals Metabolite–Protein Physical Interaction Subnetworks Altered in Cancer
Source: Mol Cell Proteomics. 2021 Dec 20;21(1):100189. doi: 10.1016/j.mcpro.2021.100189 (PMC8761777; doi:10.1016/j.mcpro.2021.100189)

Figure S1

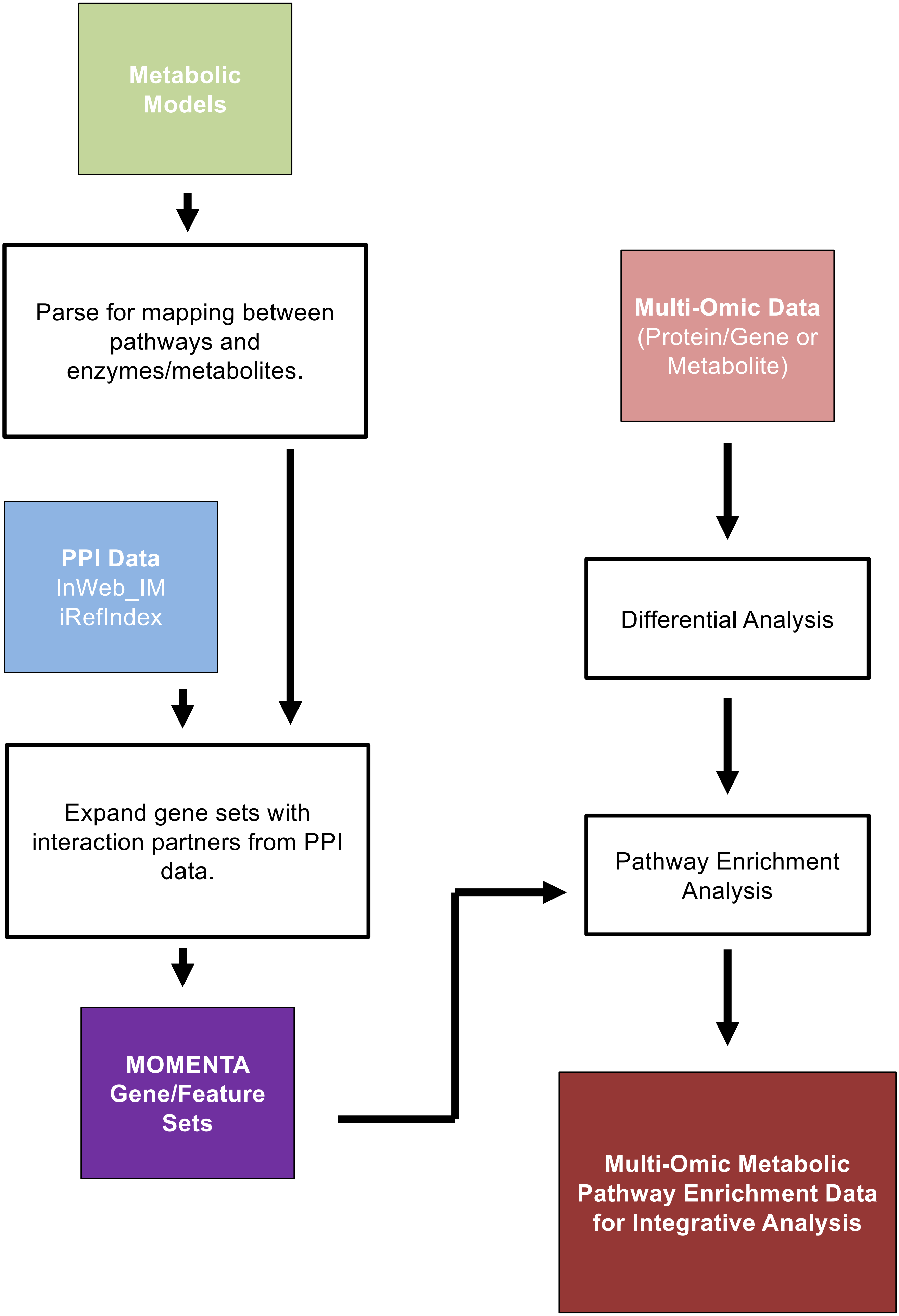

Metabolic Models
Parse for mapping between pathways and enzymes/metabolites.
PPI Data
InWeb_IM
iRefIndex
Expand gene sets with interaction partners from PPI data.
MOMENTA Gene/Feature Sets
Multi-Omic Data (Protein/Gene or Metabolite)
Differential Analysis
Pathway Enrichment Analysis
Multi-Omic Metabolic Pathway Enrichment Data for Integrative Analysis

Figure S2

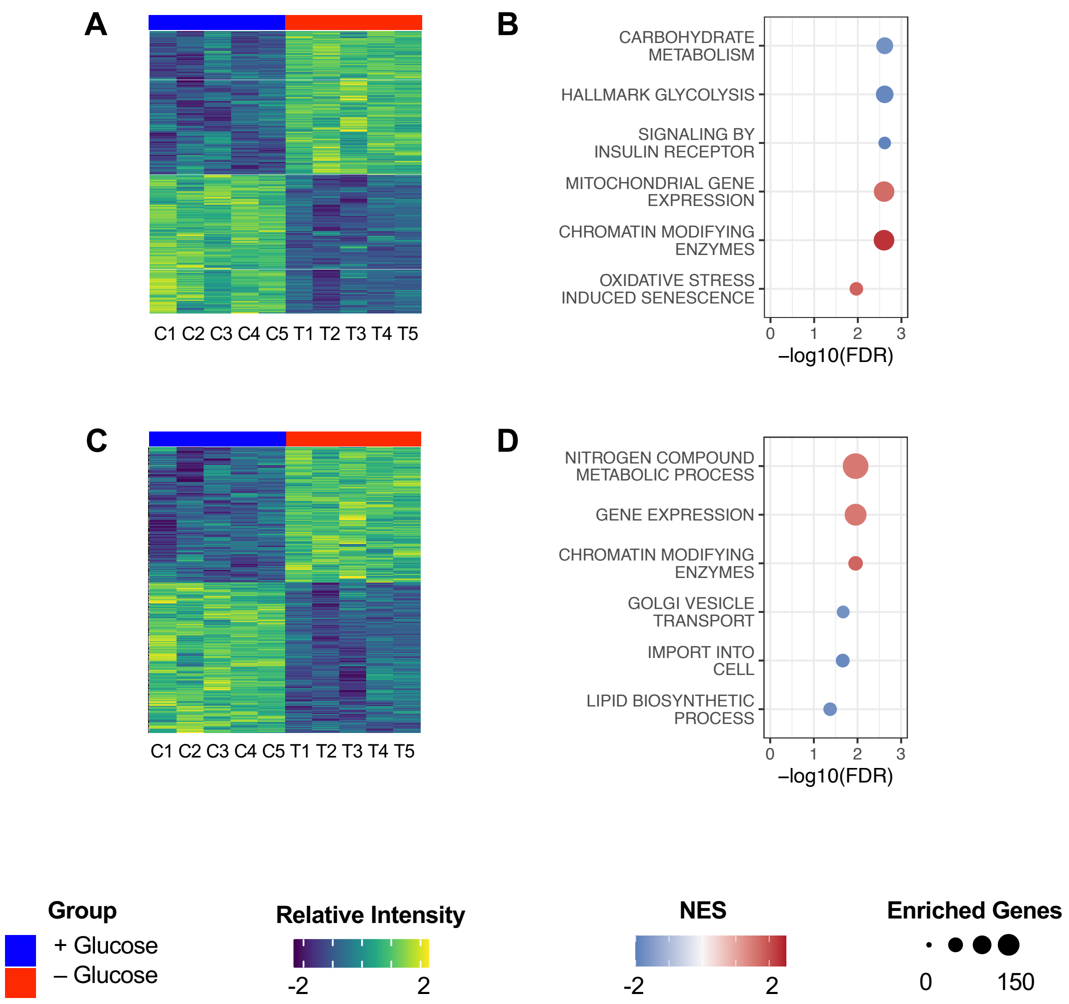
A
C1 C2 C3 C4 C5 T1 T2 T3 T4 T5
B
CARBOHYDRATE METABOLISM
HALLMARK GLYCOLYSIS
SIGNALING BY INSULIN RECEPTOR
MITOCHONDRIAL GENE EXPRESSION
CHROMATIN MODIFYING ENZYMES
OXIDATIVE STRESS INDUCED SENESCENCE
0 1 2 3
−log10(FDR)
C
C1 C2 C3 C4 C5 T1 T2 T3 T4 T5
D
NITROGEN COMPOUND METABOLIC PROCESS
GENE EXPRESSION
CHROMATIN MODIFYING ENZYMES
GOLGI VESICLE TRANSPORT
IMPORT INTO CELL
LIPID BIOSYNTHETIC PROCESS
0 1 2 3
−log10(FDR)
Group
+ Glucose
– Glucose
Relative Intensity
-2
2
NES
-2
2
Enriched Genes
0
150

Figure S3

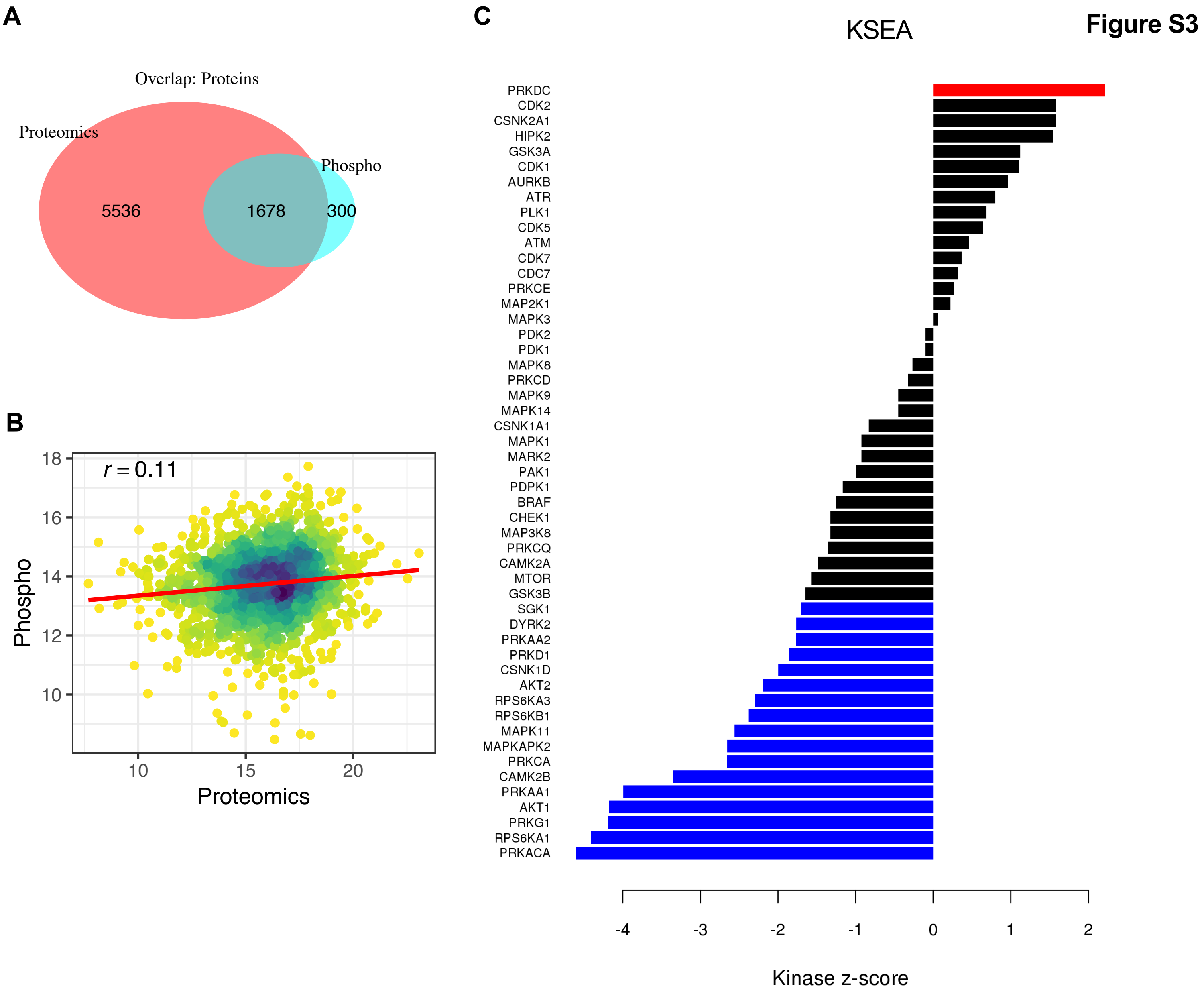
A
Overlap: Proteins
Proteomics
Phospho
5536
1678
300
B
r = 0.11
Phospho
Proteomics
18
16
14
12
10
10
15
20
C
KSEA
PRKDC
CDK2
CSNK2A1
HIPK2
GSK3A
CDK1
AURKB
ATR
PLK1
CDK5
ATM
CDK7
CDC7
PRKCE
MAP2K1
MAPK3
PDK2
PDK1
MAPK8
PRKCD
MAPK9
MAPK14
CSNK1A1
MAPK1
MARK2
PAK1
PDPK1
BRAF
CHEK1
MAP3K8
PRKCQ
CAMK2A
MTOR
GSK3B
SGK1
DYRK2
PRKAA2
PRKD1
CSNK1D
AKT2
RPS6KA3
RPS6KB1
MAPK11
MAPKAPK2
PRKCA
CAMK2B
PRKAA1
AKT1
PRKG1
RPS6KA1
PRKACA
-4
-3
-2
-1
0
1
2
Kinase z-score

Figure S4

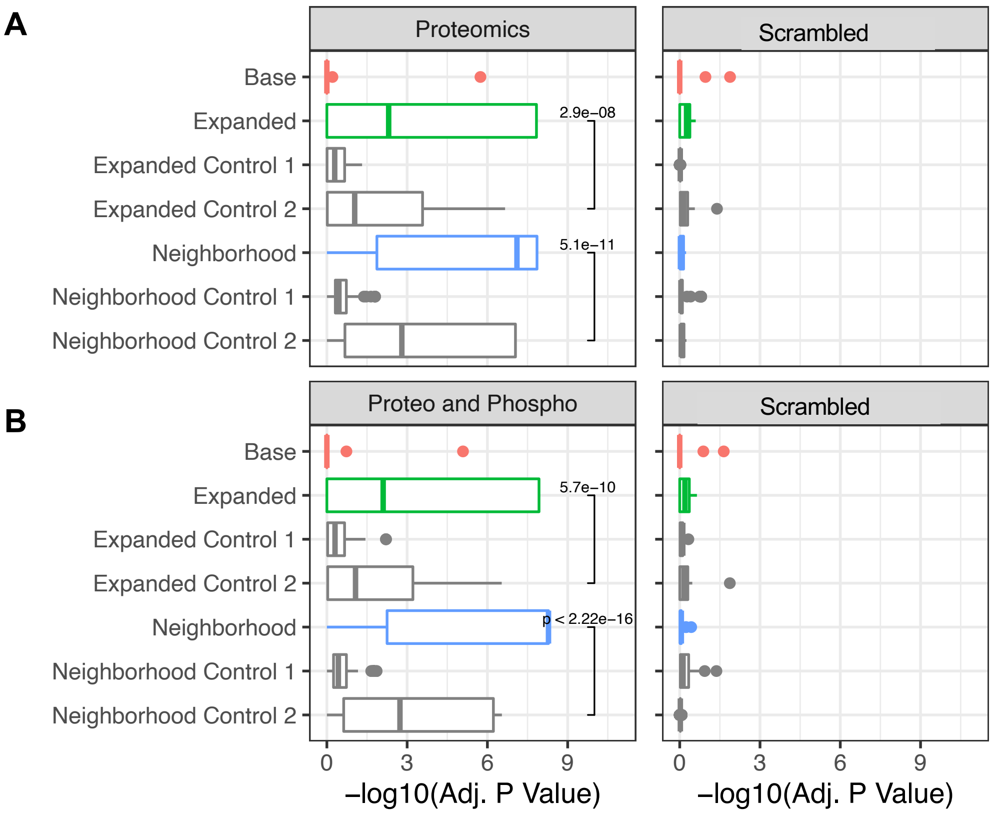
A
Proteomics
Scrambled
Base
Expanded
Expanded Control 1
Expanded Control 2
Neighborhood
Neighborhood Control 1
Neighborhood Control 2
2.9e−08
5.1e−11
B
Proteo and Phospho
Scrambled
5.7e−10
p < 2.22e−16
0 3 6 9
−log10(Adj. P Value)
−log10(Adj. P Value)

Figure S5

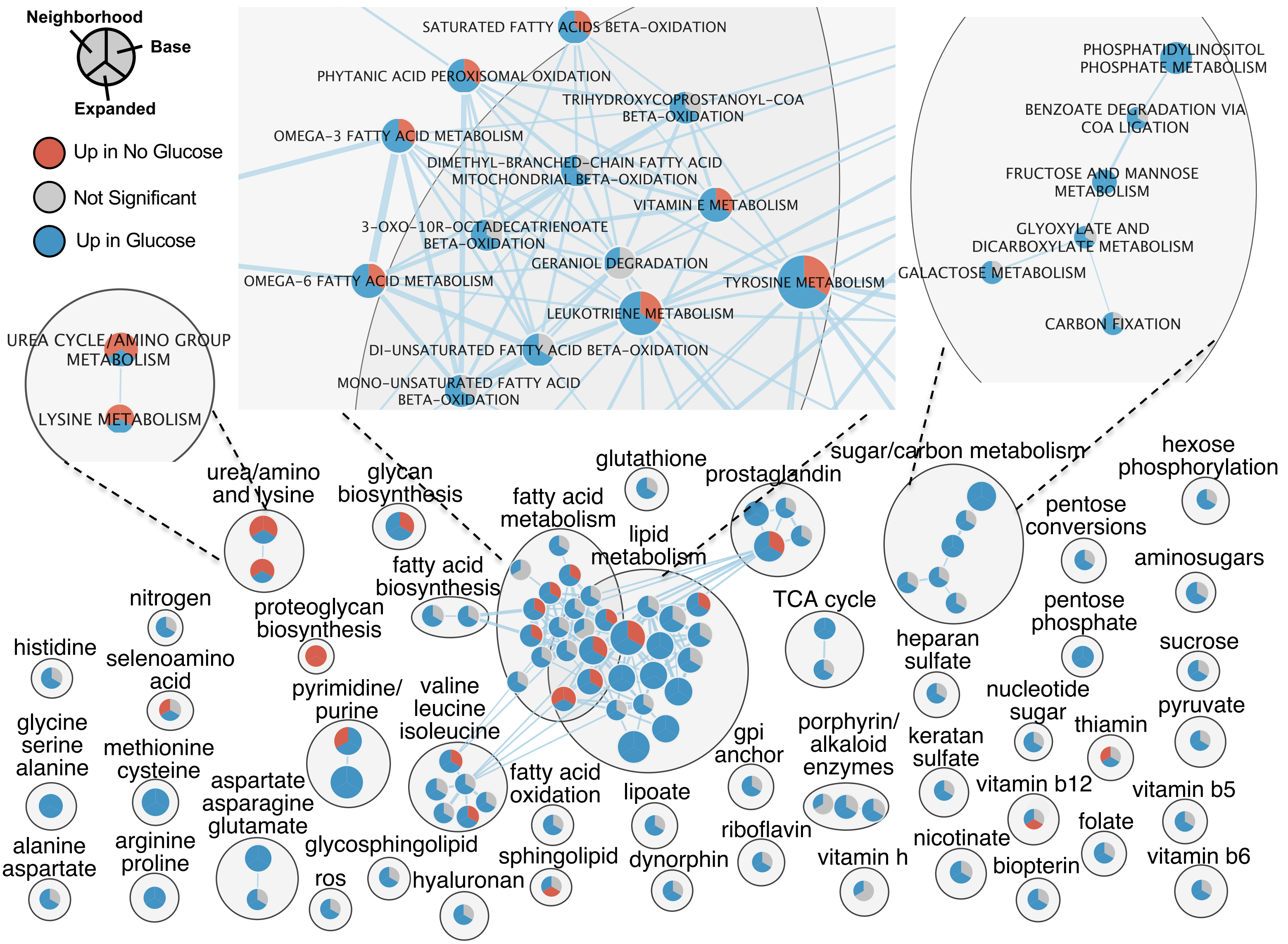

Neighborhood
Base
Expanded
Up in No Glucose
Not Significant
Up in Glucose
SATURATED FATTY ACIDS BETA-OXIDATION
PHYTANIC ACID PEROXISOMAL OXIDATION
TRIHYDROXYCOPROSTANOYL-COA BETA-OXIDATION
OMEGA-3 FATTY ACID METABOLISM
DIMETHYL-BRANCHED-CHAIN FATTY ACID MITOCHONDRIAL BETA-OXIDATION
VITAMIN E METABOLISM
3-OXO-10R-OCTADECATRIENOATE BETA-OXIDATION
GERANIOL DEGRADATION
OMEGA-6 FATTY ACID METABOLISM
TYROSINE METABOLISM
LEUKOTRIENE METABOLISM
DI-UNSATURATED FATTY ACID BETA-OXIDATION
MONO-UNSATURATED FATTY ACID BETA-OXIDATION
PHOSPHATIDYLINOSITOL PHOSPHATE METABOLISM
BENZOATE DEGRADATION VIA COA LIGATION
FRUCTOSE AND MANNOSE METABOLISM
GLYOXYLATE AND DICARBOXYLATE METABOLISM
GALACTOSE METABOLISM
CARBON FIXATION
UREA CYCLE/AMINO GROUP METABOLISM
LYSINE METABOLISM
urea/amino and lysine
glycan biosynthesis
glutathione
fatty acid metabolism
prostaglandin
sugar/carbon metabolism
hexose phosphorylation
pentose conversions
lipid metabolism
aminosugars
fatty acid biosynthesis
nitrogen
proteoglycan biosynthesis
TCA cycle
pentose phosphate
histidine
selenoamino acid
heparan sulfate
sucrose
pyrimidine/ purine
valine leucine isoleucine
nucleotide sugar
pyruvate
glycine serine alanine
gpi anchor
porphyrin/ alkaloid enzymes
keratan sulfate
thiamin
methionine cysteine
fatty acid oxidation
aspartate asparagine glutamate
lipoate
vitamin b12
vitamin b5
alanine aspartate
arginine proline
glycosphingolipid
riboflavin
folate
sphingolipid
dynorphin
vitamin h
nicotinate
vitamin b6
ros
hyaluronan
biopterin

Figure S6

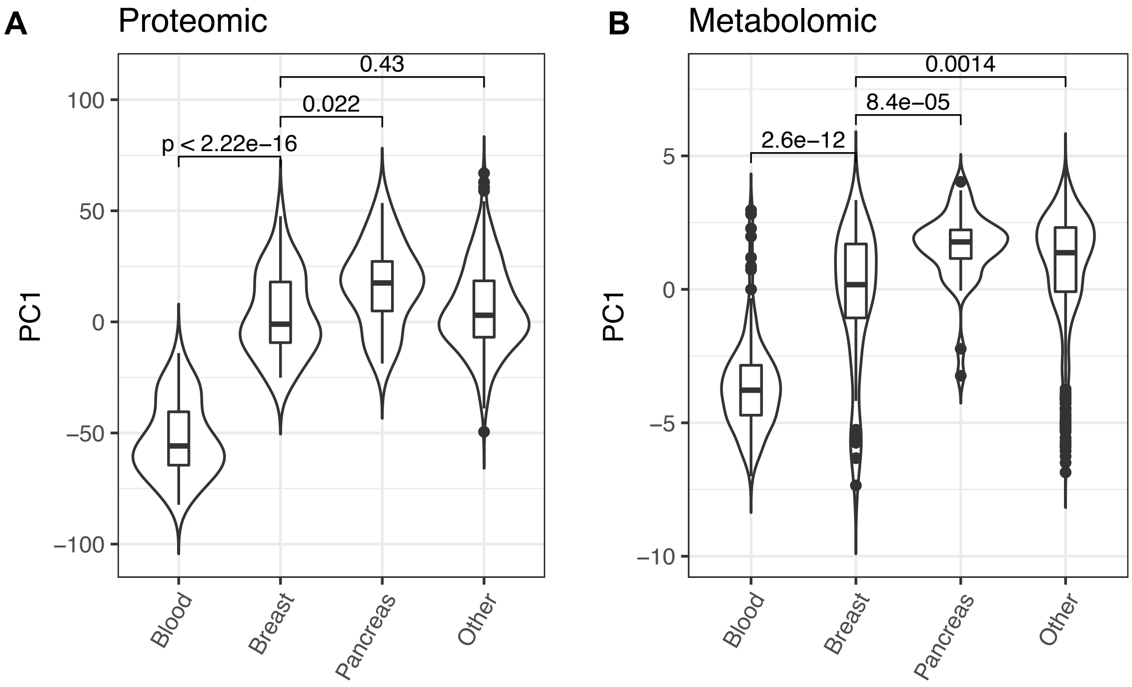
A
Proteomic
PC1
100
50
0
−50
−100
p < 2.22e−16
0.022
0.43
Blood
Breast
Pancreas
Other
B
Metabolomic
PC1
5
0
−5
−10
2.6e−12
8.4e−05
0.0014
Blood
Breast
Pancreas
Other

Figure S7

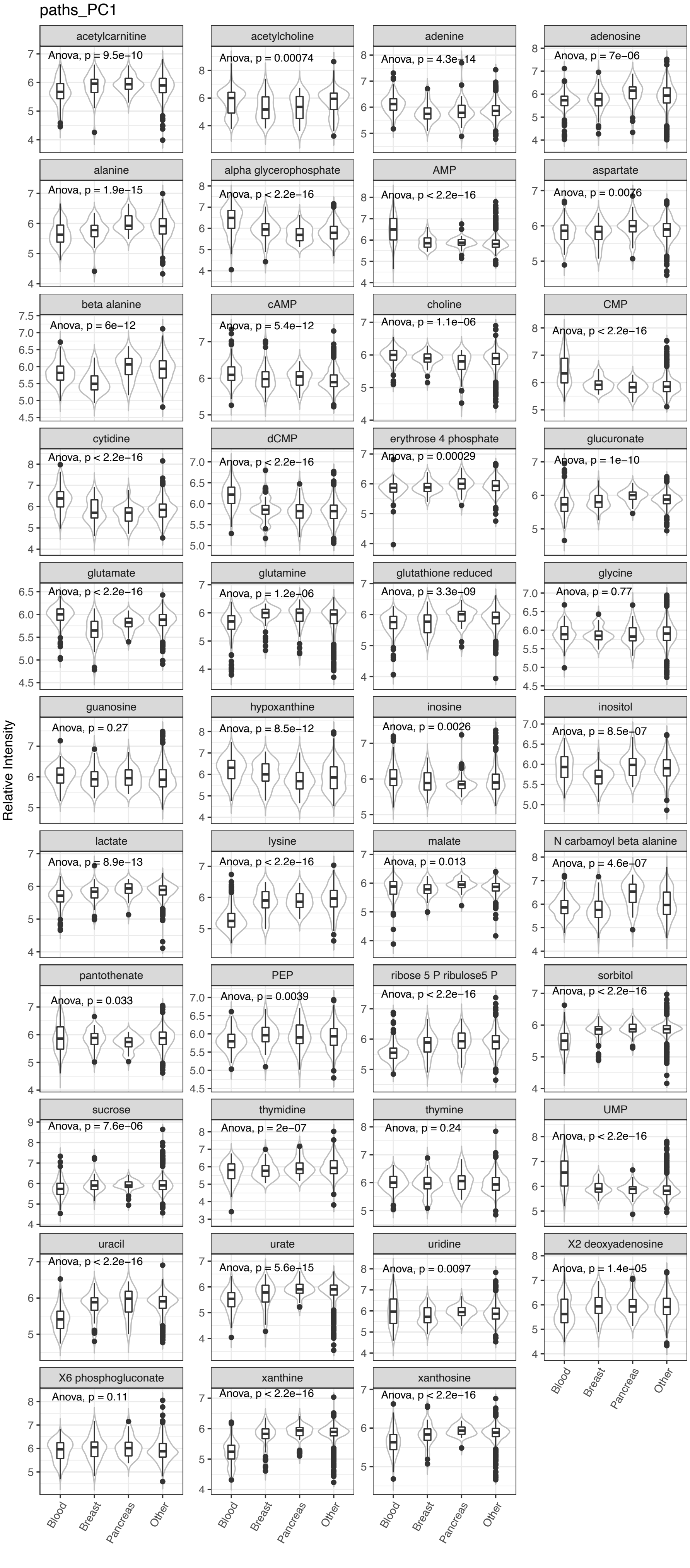
paths_PC1
Relative Intensity
acetylcarnitine
Anova, p = 9.5e−10
acetylcholine
Anova, p = 0.00074
adenine
Anova, p = 4.3e−14
adenosine
Anova, p = 7e−06
alanine
Anova, p = 1.9e−15
alpha glycerophosphate
Anova, p < 2.2e−16
AMP
Anova, p < 2.2e−16
aspartate
Anova, p = 0.0076
beta alanine
Anova, p = 6e−12
cAMP
Anova, p = 5.4e−12
choline
Anova, p = 1.1e−06
CMP
Anova, p < 2.2e−16
cytidine
Anova, p < 2.2e−16
dCMP
Anova, p < 2.2e−16
erythrose 4 phosphate
Anova, p = 0.00029
glucuronate
Anova, p = 1e−10
glutamate
Anova, p < 2.2e−16
glutamine
Anova, p = 1.2e−06
glutathione reduced
Anova, p = 3.3e−09
glycine
Anova, p = 0.77
guanosine
Anova, p = 0.27
hypoxanthine
Anova, p = 8.5e−12
inosine
Anova, p = 0.0026
inositol
Anova, p = 8.5e−07
lactate
Anova, p = 8.9e−13
lysine
Anova, p < 2.2e−16
malate
Anova, p = 0.013
N carbamoyl beta alanine
Anova, p = 4.6e−07
pantothenate
Anova, p = 0.033
PEP
Anova, p = 0.0039
ribose 5 P ribulose5 P
Anova, p < 2.2e−16
sorbitol
Anova, p < 2.2e−16
sucrose
Anova, p = 7.6e−06
thymidine
Anova, p = 2e−07
thymine
Anova, p = 0.24
UMP
Anova, p < 2.2e−16
uracil
Anova, p < 2.2e−16
urate
Anova, p = 5.6e−15
uridine
Anova, p = 0.0097
X2 deoxyadenosine
Anova, p = 1.4e−05
X6 phosphogluconate
Anova, p = 0.11
xanthine
Anova, p < 2.2e−16
xanthosine
Anova, p < 2.2e−16
Blood
Breast
Pancreas
Other

Figure S8

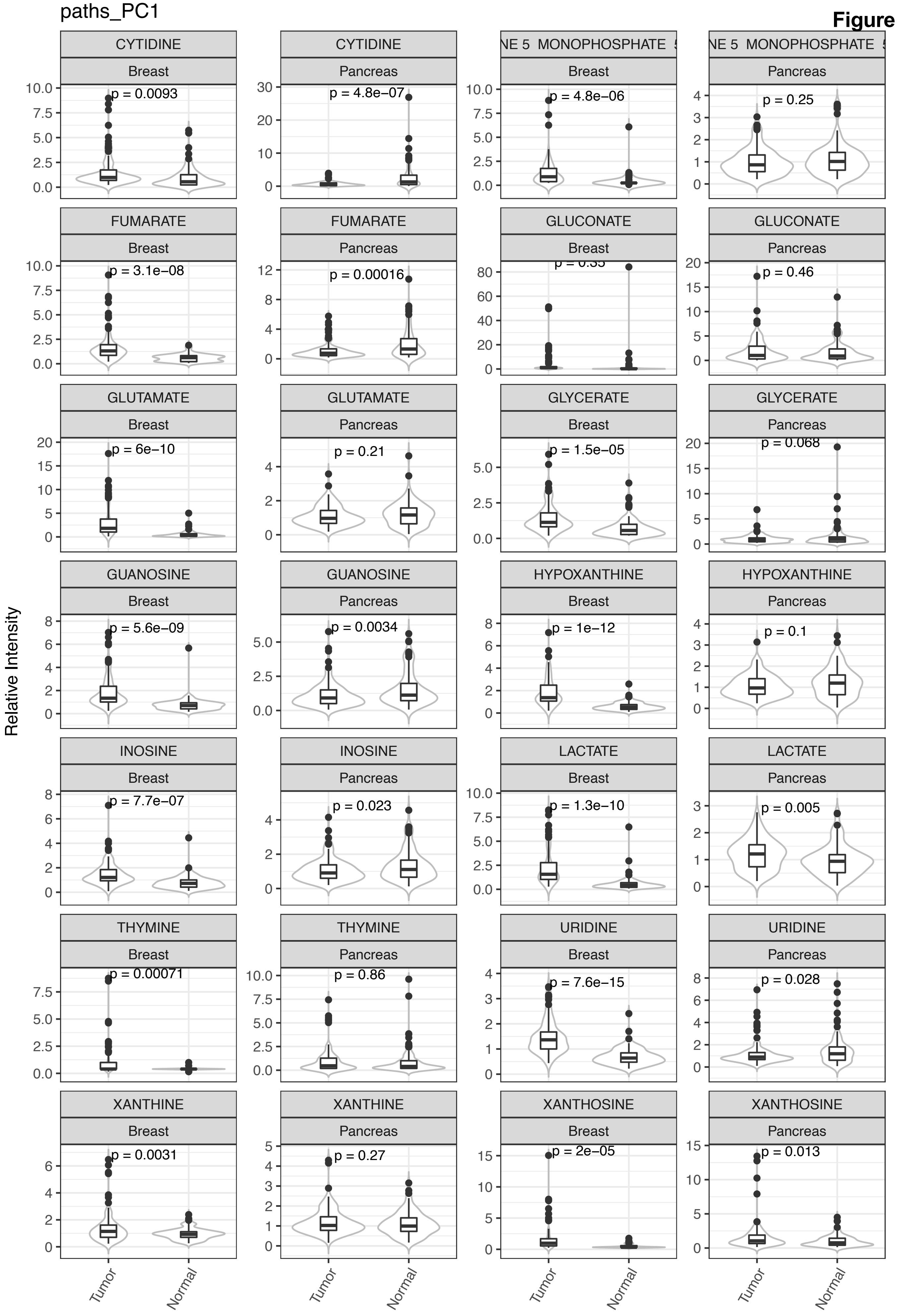
paths_PC1
Relative Intensity
CYTIDINE
Breast
p = 0.0093
CYTIDINE
Pancreas
p = 4.8e−07
NE 5 MONOPHOSPHATE
Breast
p = 4.8e−06
NE 5 MONOPHOSPHATE
Pancreas
p = 0.25
FUMARATE
Breast
p = 3.1e−08
FUMARATE
Pancreas
p = 0.00016
GLUCONATE
Breast
GLUCONATE
Pancreas
p = 0.46
GLUTAMATE
Breast
p = 6e−10
GLUTAMATE
Pancreas
p = 0.21
GLYCERATE
Breast
p = 1.5e−05
GLYCERATE
Pancreas
p = 0.068
GUANOSINE
Breast
p = 5.6e−09
GUANOSINE
Pancreas
p = 0.0034
HYPOXANTHINE
Breast
p = 1e−12
HYPOXANTHINE
Pancreas
p = 0.1
INOSINE
Breast
p = 7.7e−07
INOSINE
Pancreas
p = 0.023
LACTATE
Breast
p = 1.3e−10
LACTATE
Pancreas
p = 0.005
THYMINE
Breast
p = 0.00071
THYMINE
Pancreas
p = 0.86
URIDINE
Breast
p = 7.6e−15
URIDINE
Pancreas
p = 0.028
XANTHINE
Breast
p = 0.0031
XANTHINE
Pancreas
p = 0.27
XANTHOSINE
Breast
p = 2e−05
XANTHOSINE
Pancreas
p = 0.013
Tumor
Normal

Figure S9

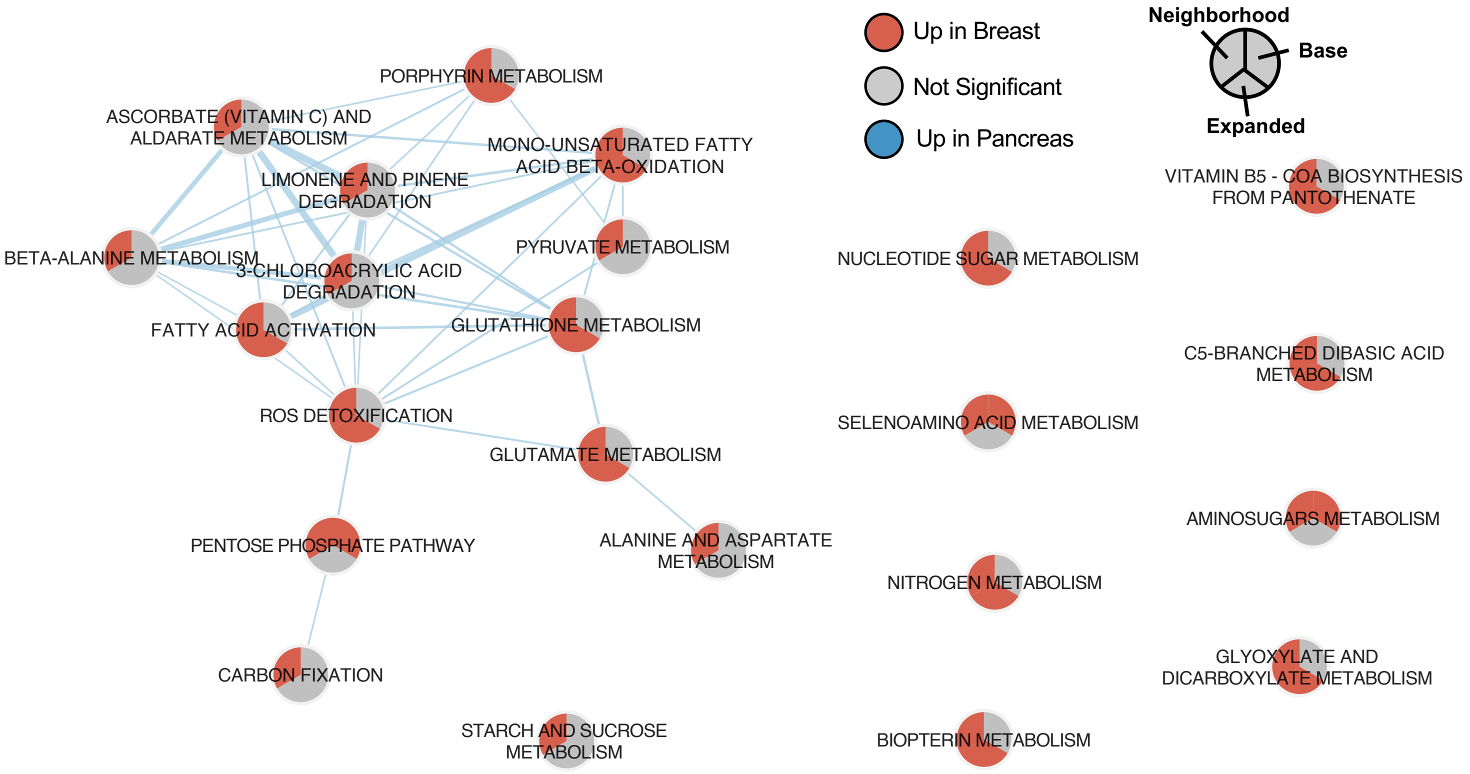
Up in Breast
Not Significant
Up in Pancreas
Neighborhood
Base
Expanded
PORPHYRIN METABOLISM
ASCORBATE (VITAMIN C) AND ALDARATE METABOLISM
MONO-UNSATURATED FATTY ACID BETA-OXIDATION
LIMONENE AND PINENE DEGRADATION
BETA-ALANINE METABOLISM
3-CHLOROACRYLIC ACID DEGRADATION
PYRUVATE METABOLISM
FATTY ACID ACTIVATION
GLUTATHIONE METABOLISM
ROS DETOXIFICATION
GLUTAMATE METABOLISM
PENTOSE PHOSPHATE PATHWAY
ALANINE AND ASPARTATE METABOLISM
CARBON FIXATION
STARCH AND SUCROSE METABOLISM
VITAMIN B5 - COA BIOSYNTHESIS FROM PANTOTHENATE
NUCLEOTIDE SUGAR METABOLISM
C5-BRANCHED DIBASIC ACID METABOLISM
SELENOAMINO ACID METABOLISM
AMINOSUGARS METABOLISM
NITROGEN METABOLISM
GLYOXYLATE AND DICARBOXYLATE METABOLISM
BIOPTERIN METABOLISM

Figure S10

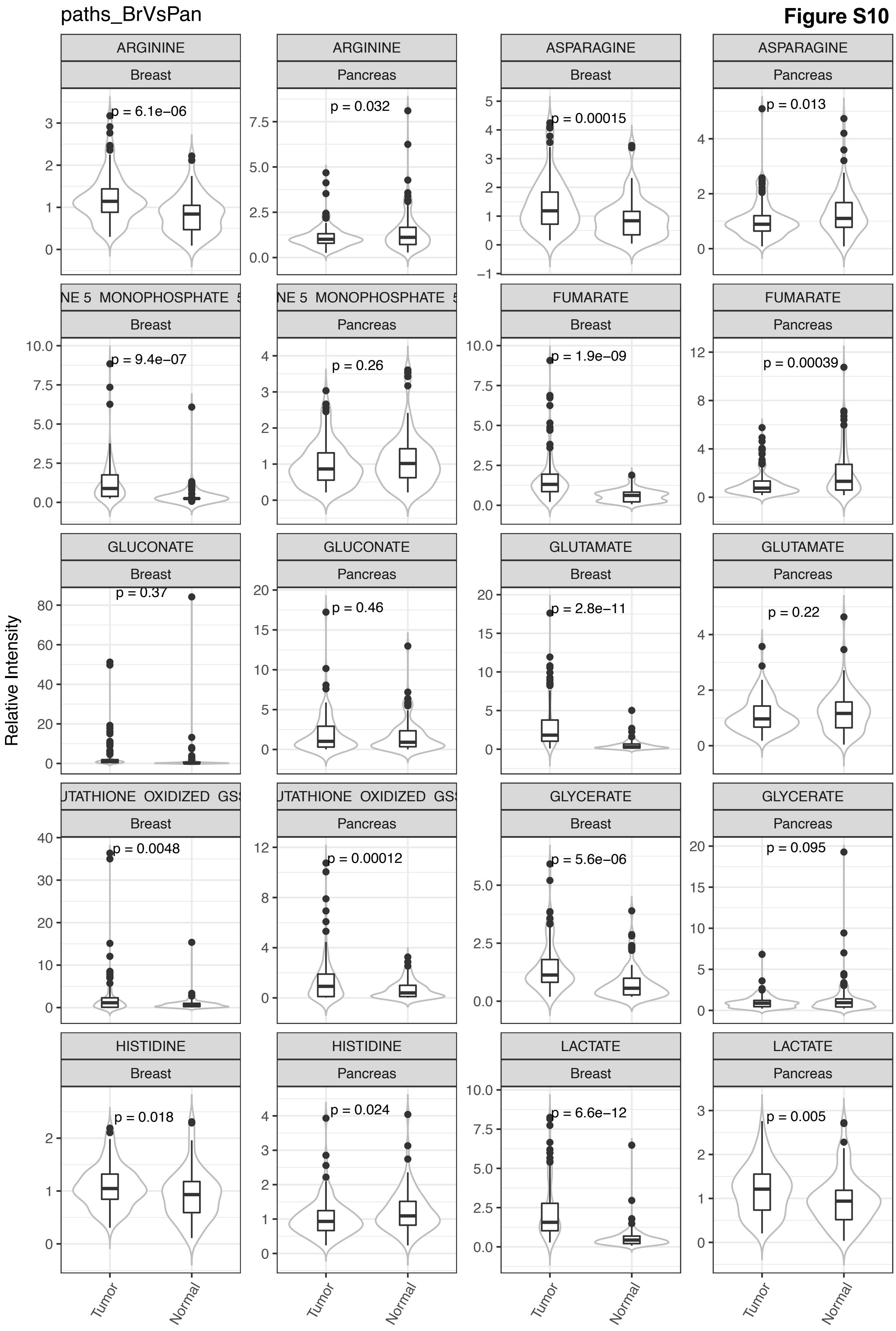
paths_BrVsPan
ARGININE
Breast
p = 6.1e−06
ARGININE
Pancreas
p = 0.032
ASPARAGINE
Breast
p = 0.00015
ASPARAGINE
Pancreas
p = 0.013
NE 5 MONOPHOSPHATE 5
Breast
p = 9.4e−07
NE 5 MONOPHOSPHATE 5
Pancreas
p = 0.26
FUMARATE
Breast
p = 1.9e−09
FUMARATE
Pancreas
p = 0.00039
GLUCONATE
Breast
p = 0.37
GLUCONATE
Pancreas
p = 0.46
GLUTAMATE
Breast
p = 2.8e−11
GLUTAMATE
Pancreas
p = 0.22
UTATHIONE OXIDIZED GS
Breast
p = 0.0048
UTATHIONE OXIDIZED GS
Pancreas
p = 0.00012
GLYCERATE
Breast
p = 5.6e−06
GLYCERATE
Pancreas
p = 0.095
HISTIDINE
Breast
p = 0.018
HISTIDINE
Pancreas
p = 0.024
LACTATE
Breast
p = 6.6e−12
LACTATE
Pancreas
p = 0.005
Relative Intensity
Tumor
Normal

Pan Cancer Metabolism Data
Lysine and Urea Cycle/Amino Group Pathway Metabolites

Figure S11

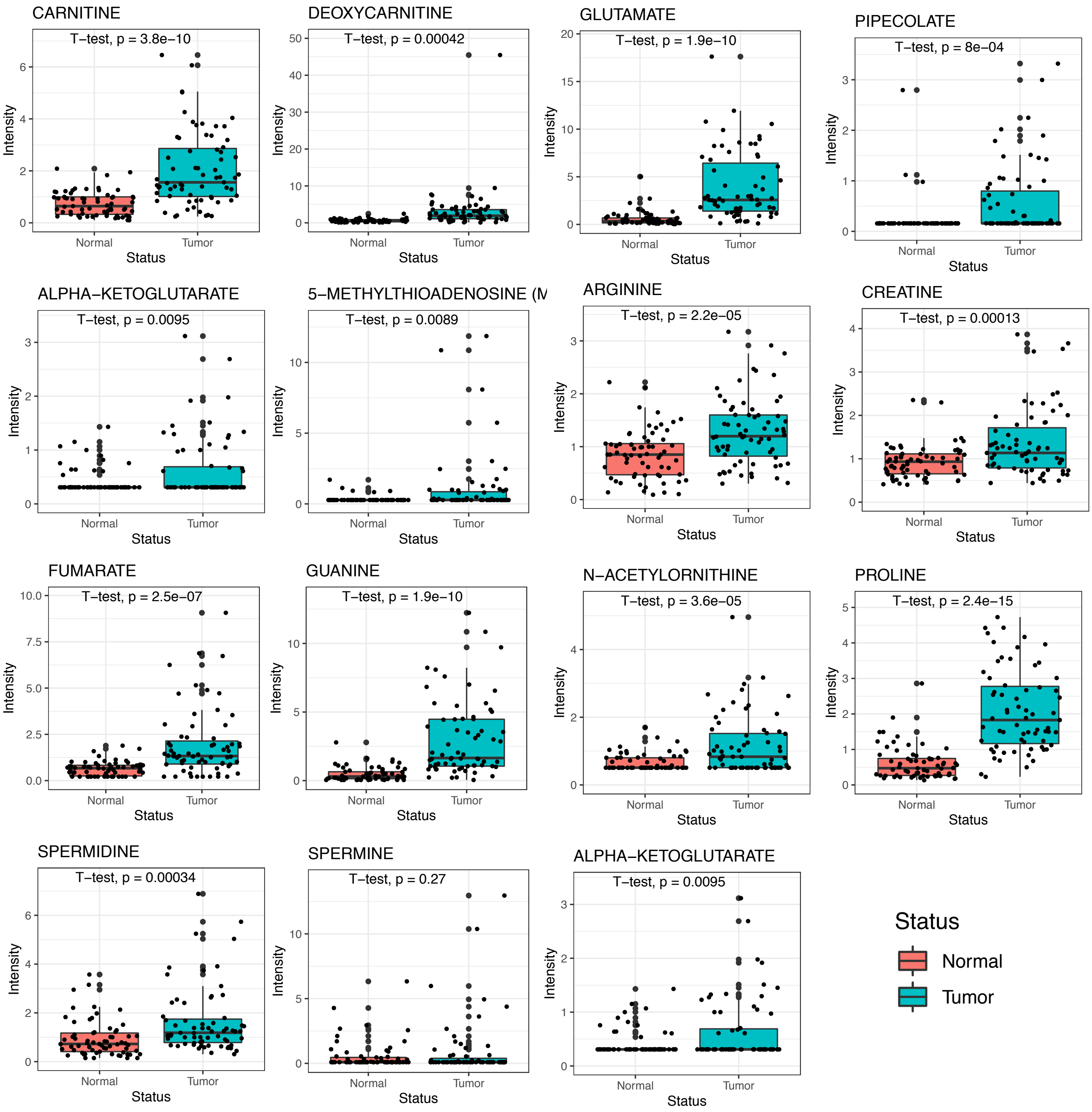

Pan Cancer Metabolism Data
Lysine and Urea Cycle/Amino Group Pathway Metabolites

Figure S12

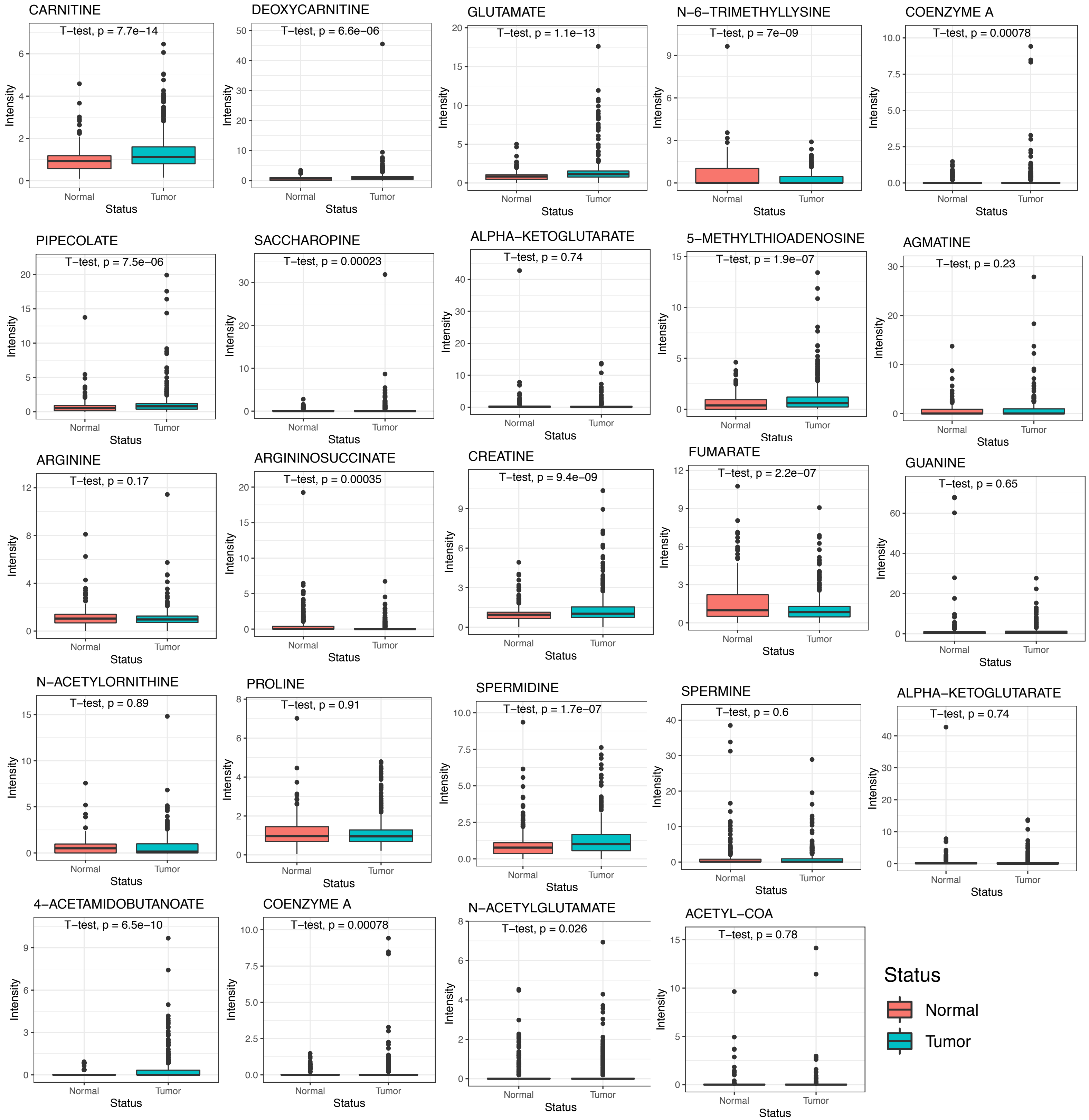

Supplement: Supplemental Figures S1–S12 [file mmc1.pdf]
