# Supplementary material for: Multiomic Metabolic Enrichment Network Analysis Reveals Metabolite–Protein Physical Interaction Subnetworks Altered in Cancer
Source: Mol Cell Proteomics. 2021 Dec 20;21(1):100189. doi: 10.1016/j.mcpro.2021.100189 (PMC8761777; doi:10.1016/j.mcpro.2021.100189)
Supplement: Supplemental Information [file mmc2.docx]

**SUPPLEMENTARY INFORMATION**

**Figure S1**. Workflow of MOMENTA integration and data processing. First, metabolic models are parsed to obtain pathway information (enzymes and other primary interacting proteins), which is then expanded with data from protein-protein interaction (PPI) databases to increase protein mapping to pathways (left). In parallel, proteomic and/or metabolomic data are analyzed through differential analysis (right). The proteomic and metabolomic data is then put through an enrichment analysis step using the MOMENTA gene sets to interrogate metabolic pathways and integrate the data at the level of biochemical pathways.

**Figure S2.** Additional analysis of glucose starved cancer cell line. ***A***, Differential analysis of proteomic data showing top differential proteins across all replicates. ***B***, Enrichment results of selected pathways for proteomic data from gene set enrichment analysis with large, curated gene set library. ***C***, Differential analysis of phosphoproteomic data showing top differential proteins across all replicates. ***D***, Enrichment results from phosphoproteomic data of selected pathways from gene set enrichment analysis with large, curated gene set library.

**Figure S3.** Additional coverage provided by incorporating phosphoproteomic analysis. **A**, Venn Diagram of proteins detected in proteomics data and phosphoproteomics data. **B**, Correlation of average intensity values across proteomics data and phosphoproteomics data. **C**, Kinase substrate enrichment analysis of phosphoproteomic data.

**Figure S4.** Pathway enrichment results for proteomics data across MOMENTA gene sets using Biocyc metabolic model. **A**, Boxplot and individual pathway enrichment significance for all metabolic pathways in the human Biocyc model using the cancer cell line proteomic data alone and scrambled proteomic data. **B**, Boxplot and individual pathway enrichment significance for all metabolic pathways in the human Biocyc model using the cancer cell line proteomic and phosphoproteomic combined data and scrambled combined data. Significance of pathway enrichment results are shown for the base (enzyme only) gene sets, as well as the Expanded and Neighborhood gene sets. Matched control gene sets with the same number of genes from the PPI added at random (Control 1) or same number of genes from an alternative area in the network (all second-degree neighbors, Control 2).

**Figure S5.** Pathway enrichment results for phospho/proteomic data across gene sets. Network representation of significant (Adjusted P Value < 0.05) metabolic pathways enrichment results from the *Base, Expanded,* and *Neighborhood* gene sets. Pathways (nodes) are connected by edges based on overlapping features to group into functionally related families. Nodes are split into thirds with the top right corner representing the *Base* enrichment results, bottom representing the *Expanded* enrichment results, and top left representing *Neighborhood* enrichment results. The thirds are colored red for pathways up-regulated in the No Glucose condition and blue for the pathways downregulated. Pathways groups are annotated with biological themes.

**Figure S6.** Proteomic and metabolomic variation across cancer cell lines. **A,** Distribution of cancer cell lines based on tissue of origin (Blood, Breast, Pancreas, Other) along principal component 1 (PC1) from proteomic profiling of cancer cell lines. **B**, Distribution of cancer cell lines based on tissue of origin (Blood, Breast, Pancreas, Other) along principal component 1 (PC1) from metabolomic profiling of cancer cell lines. P-values for comparisons indicated based on Kruskal-Wallis test.

**Figure S7.** Metabolites from CCLE metabolomic profiling that map to significant pathways in MOMENTA *Neighborhood* PC1 enrichment analysis. Relative intensities are plotted for cancer cell lines broken out by tissue of origin categories (Blood, Breast, Pancreas, Other) and a results of an Anova test are shown.

**Figure S8.** Metabolites from Pan Cancer Metabolism Data clinical profiling of tumor and adjacent normal samples. Metabolites map to significant pathways in MOMENTA *Neighborhood* PC1 enrichment analysis. Relative intensities are plotted for each metabolite in tumor and normal for selected cancer types (Breast and Pancreas). Results of T-test shown.

**Figure S9.** Pathway enrichment results for CCLE protemeomic data comparing Breast and Pancreas derived cancer cell lines. Network representation of significant (Adjusted P Value < 0.05) metabolic pathways enrichment results from the *Base, Expanded,* and *Neighborhood* gene sets. Pathways (nodes) are connected by edges based on overlapping features to group into functionally related families. Nodes are split into thirds with the top right corner representing the *Base* enrichment results, bottom representing the *Expanded* enrichment results, and top left representing *Neighborhood* enrichment results. The thirds are colored red for pathways up-regulated in the Breast cancer lines.

**Figure S10.** Metabolites from Pan Cancer Metabolism Data clinical profiling of tumor and adjacent normal samples. Metabolites map to significant pathways in MOMENTA *Neighborhood* Breast cancer cell lines vs Pancreas cancer cell lines enrichment analysis. Relative intensities are plotted for each metabolite in tumor and normal for selected cancer types (Breast and Pancreas). Results of T-test shown.

**Figure S11.** Boxplot and individual metabolite intensities from the Pan Cancer Metabolism Data for breast cancer across tumor and normal samples for metabolites in the Lysine and Urea Cycle/Amino Group metabolism pathways. Results of T-test shown.

**Figure S12.** Boxplots of metabolite intensities from the Pan Cancer Metabolism Data for all cancer types across tumor and normal samples. Showing metabolites in the Lysine and Urea Cycle/Amino Group metabolism pathways. Results of T-test shown.

**Table S1.** Feature sizes from metabolic models downloaded for integrative enrichment analysis. Features include metabolites, enzymes (E.C. codes), genes, and genes after expansion with protein-protein interaction (PPI) data using the expanded and neighborhood (target size) methods.

**Table S2.** Feature set sizes for each pathway in the Human MFN metabolic model. Feature numbers are shown for each pathway including metabolites, enzymes (E.C. codes), genes, as well as the expanded and neighborhood gene sets.

**Table S3A.** Log-transformed proteomic (protein groups) TMT reporter ion intensities following normalization and filtering for cancer cell line data with gene and protein identity annotation.

**Table S3B.** MaxQuant proteinGroups results table.

**Table S4A.** Log-transformed phosphosite TMT reporter ion intensities following normalization and filtering for cancer cell line data with residue, gene, and protein identity annotation.

**Table S4B.** MaxQuant Phospho Sites (S, T, Y) results table.

**Table S5.** Gene set enrichment analysis (GSEA) for cancer cell line proteomic data using large, curated library of gene sets.

**Table S6.** Gene set enrichment analysis (GSEA) for cancer cell line phosphosite data using large, curated library of gene sets.

**Table S7.** Combined proteomic and phosphoproteomic enrichment results using Base gene sets and fgsea R package for cancer cell line data.

**Table S8.** Combined proteomic and phosphoproteomic enrichment results using Expanded gene sets and fgsea R package for cancer cell line data.

**Table S9.** Combined proteomic and phosphoproteomic enrichment results using Neighborhood gene sets and fgsea R package for cancer cell line data.

**Table S10.** Metabolic pathway enrichment results using MetaboAnalyst for cancer cell line metabolomic data.

**Table S11.** MOMENTA-based *Neighborhood* enrichment analysis for CCLE proteomic profiling of cancer cell lines.

**Table S12.** MOMENTA-based *Neighborhood* enrichment analysis for CPTAC proteomic profiling of tumor and adjacent normal samples.
